# CRISPR-Cas immunity leads to a coevolutionary arms race between *Streptococcus thermophilus* and lytic phage

**DOI:** 10.1098/rstb.2018.0098

**Published:** 2019-03-25

**Authors:** Jack Common, Daniel Morley, Edze R. Westra, Stineke van Houte

**Affiliations:** ESI and CEC, Biosciences, University of Exeter, Cornwall Campus, Penryn TR10 9EZ, UK

**Keywords:** CRISPR-Cas, host–parasite coevolution, arms race, *Streptococcus thermophilus*, phage

## Abstract

CRISPR-Cas is an adaptive prokaryotic immune system that prevents phage infection. By incorporating phage-derived ‘spacer’ sequences into CRISPR loci on the host genome, future infections from the same phage genotype can be recognized and the phage genome cleaved. However, the phage can escape CRISPR degradation by mutating the sequence targeted by the spacer, allowing them to re-infect previously CRISPR-immune hosts, and theoretically leading to coevolution. Previous studies have shown that phage can persist over long periods in populations of *Streptococcus thermophilus* that can acquire CRISPR-Cas immunity, but it has remained less clear whether this coexistence was owing to coevolution, and if so, what type of coevolutionary dynamics were involved. In this study, we performed highly replicated serial transfer experiments over 30 days with *S. thermophilus* and a lytic phage. Using a combination of phenotypic and genotypic data, we show that CRISPR-mediated resistance and phage infectivity coevolved over time following an arms race dynamic, and that asymmetry between phage infectivity and host resistance within this system eventually causes phage extinction. This work provides further insight into the way CRISPR-Cas systems shape the population and coevolutionary dynamics of bacteria–phage interactions.

This article is part of a discussion meeting issue ‘The ecology and evolution of prokaryotic CRISPR-Cas adaptive immune systems’.

## 1. Introduction

Clustered regularly interspaced short palindromic repeats and their associated *cas* genes (CRISPR-Cas) form an adaptive immune system that is found in approximately 50% of all bacteria and 90% of archaea [1]. CRISPR-Cas confers immunity to phage infection by incorporating phage-derived sequences into CRISPR loci on the host genome. These loci consist of repeating sequences (repeats) that are interspaced by sequences (spacers) derived from phage and other mobile genetic elements of typically around 30 nt in length. RNA transcripts of CRISPR loci are processed and form a ribonucleoprotein complex with Cas proteins that can recognize and cleave complementary nucleic acid sequences, preventing future infections by the same phage genotype. CRISPR-Cas systems are highly diverse and are currently ordered into two classes, six types and 33 subtypes based on their *cas* gene composition, gene synteny and CRISPR repeat sequences, with clear differences in the molecular mechanisms of different variants [2].

In some natural environments, bacteria with CRISPR-Cas systems appear to coevolve with phage over long time periods [3,4]. However, studying the dynamics of these coevolutionary interactions under controlled laboratory conditions has been limited by the availability of adequate model systems. Specifically, while many bacteria encode CRISPR-Cas immune systems, under laboratory conditions, the vast majority do not evolve CRISPR-based immunity upon phage or plasmid infection or do so at such low frequencies that they are detectable only with deep-sequencing approaches. Such low-frequency CRISPR evolution is unlikely to significantly contribute to the reciprocal selection between the bacteria and the phage. Currently, only two bacterial species have been found to naturally evolve (almost) exclusively CRISPR-based immunity under laboratory conditions: *Streptococcus thermophilus* strains DGCC7710 and LMD-9 [5–7], and *Pseudomonas aeruginosa* strain UCBPP-PA14 [8].

Early studies with *S. thermophilus* demonstrated that phage can overcome CRISPR immunity by evolving point mutations in the sequence targeted by the spacer (the ‘protospacer’), or in the protospacer-adjacent motif (PAM) [5], a conserved sequence immediately adjacent to the protospacer that is used by the bacteria to discriminate between self (i.e. CRISPR loci) and non-self (i.e. phage) DNA [9–11]. This observation suggested a possible scenario for coevolution in free-running systems, where bacteria acquire spacers over time and phage escape via point mutations in the corresponding protospacers or PAMs [12–14]. Consistent with this idea, it was reported that *S. thermophilus* can coexist with phage over many generations and that for each treatment the single experimental population displayed large fluctuations in its spacer repertoire and an increase in the frequency of point mutations in phage genomes over time [15–17]. However, a more recent study suggested that coevolution is unable to explain long-term coexistence of *S. thermophilus* and its phage, suggesting instead that this may be driven by a back mutation of bacteria with CRISPR immunity to sensitive phenotypes [18], which would provide a continuous supply of sensitive hosts for the phage to amplify in. Such loss of CRISPR immunity owing to mutation has also been observed at high frequencies in *Staphylococcus epidermidis* [19], and reversion to sensitive phenotypes more generally may be an important mechanism for bacteria–phage coexistence [20].

Given the lack of clarity surrounding the role, if any, and the type of CRISPR–phage coevolution for bacteria–phage coexistence, we performed highly replicated, long-term (30-day) serial transfer experiments with *S. thermophilus* and its lytic phage 2972. Our phenotypic assays demonstrate that bacteria and phage coevolved in these experiments during at least the first 9 days (approximately 70 generations). We next examined the type of coevolutionary dynamics during this period, with a clear distinction between fluctuating selection dynamics (FSD), where the rare host and pathogen genotypes are favoured through negative frequency-dependent selection, and arms race dynamics (ARD), where host resistance and phage infectivity increase over time [21]. We found that CRISPR-mediated immunity and phage infectivity increase over time. Further, our genotypic data show that patterns of resistance and infectivity were explained by bacteria acquiring novel spacers against the phage, and the phage evolving mutations in the regions targeted by the spacers. Collectively, the data show that coevolution is probably an important factor in the coexistence of bacteria and phage in this empirical system and that this coevolution is characterized by an ARD.

## 2. Experimental methods

### (a) Strains used in the study

We used the lactic acid bacterium *S. thermophilus* DGCC7710 wild-type (WT) and its lytic *pac*-type phage 2972 (GenBank: NC_007019.1) [22] as a model system. DGCC7710 has four CRISPR-Cas systems, two of which (CRISPR1 and CRISPR3) are active during infection with phage 2972 and both are classified as type II-A [7,23]. CRISPR1 has 32 spacers and CRISPR3 has 12 spacers [7,24], none of which are perfectly complementary to a PAM-flanked sequence in the phage 2972 genome.

### (b) Phage 2972 amplification

An overnight culture of *S. thermophilus* was transferred 1 : 10 into fresh LM17 medium (M17 broth supplemented with 0.5% α-lactose) containing 10 mM CaCl_2_ and incubated shaking at 180 revolutions per minute (rpm) at 42°C. When the culture reached log phase (OD_600_ ∼ 0.25) approximately 10^6^ plaque-forming units (PFU) of phage 2972 were added and the culture was incubated under the same conditions for 2 h, at which point cells had fully lysed. Lysates were centrifuged and filtered through a 0.22 µm filter, and the resulting phage stocks were stored at 4°C.

### (c) Long-term co-culture experiment

Prior to commencing the experiment, *S. thermophilus* was acclimatized in LM17 medium at 42°C and 180 rpm for 2 days, with a 1 : 100 transfer into fresh LM17 after 24 h (approx. 10^6^ colony forming units (CFU)). To start the co-culture experiment, overnight cultures of the bacteria were transferred 1 : 100 into 6 ml LM17 media supplemented with 10 mM CaCl_2_ in glass vials. They were then infected with either 10^9^, 10^8^, 10^7^ or 10^6^ PFU of phage 2972, with 12 independent replicate experiments per treatment, followed by incubation at 42°C while shaking at 180 rpm. Replicates were transferred 1 : 100 into fresh LM17 + 10 mM CaCl_2_ every 24 h and phage titres and bacterial densities were measured every 24 h for a period of 30 days, or until no phage was detected for four consecutive days. Bacterial densities were determined through plating and colony counts, while phage densities were measured by plaque assays. These were performed by mixing phage dilutions with WT bacteria in soft agar overlays (LM17 + 10 mM CaCl_2_ and 0.5% agar), poured onto hard agar (LM17 + 10 mM CaCl_2_ and 1.5% agar).

### (d) Phage survival

Phage survival and mean time to extinction over the course of the experiment were analysed using a Cox proportional hazards model from the survival package [25].

### (e) Measuring the evolution of infectivity and resistance

To measure whether host resistance and phage infectivity evolved during their co-culture, we isolated phage clones and bacterial clones from the treatment where bacteria were infected with 10^8^ PFU phage. To give sufficient power in this analysis, we focused on eight replicate experiments from this treatment where phage persisted for at least 9 days, a period which we estimated to be sufficiently long for significant coevolution to take place. Phage extracted from 1, 4 and 9 days post-infection (dpi) were subjected to plaque assays as described above. Three time points were chosen as a minimal requirement for the downstream time-shift analysis to monitor whether coevolution took place and to determine the coevolutionary dynamics (see below). For each replicate and time point, 12 plaques were randomly picked and amplified in 96-well plates containing LM17 + 10 mM CaCl_2_ in which WT bacteria were inoculated 1 : 100 from a fresh overnight culture. Bacteria extracted from the same time points were diluted and plated overnight, and 12 colonies from each replicate were picked at random and used to make soft agar overlays on LM17 agar lawns. To examine the evolution of phage infectivity for each of the eight replicates, the 36 phage clones that were isolated (12 phage clones × 3 time points) were stamped using a 96-pin replicator onto 36 bacterial lawns corresponding to the bacterial clones isolated from the same replicate (i.e. 12 bacterial clones × 3 time points). Phage were classified as being infective against a particular bacterial clone if a clear lysis zone was visible on the lawn after incubation at 42°C for 24 h. If no lysis zone was visible, the host was classified as resistant.

### (f) Evolution of infectivity and resistance

Using the data from the experiments described above, we measured the evolution of phage infectivity as the proportion of bacterial clones that phage from each time point from the same replicate experiment could infect (i.e. how phage infectivity range changed over time). In a similar way, we measured the evolution of host resistance as the proportion of all phage genotypes from the same replicate experiment that could be resisted by bacteria from each time point (i.e. how host resistance range changed over time). Infectivity or resistance was analysed in a generalized linear model (GLM) with genotype as a fixed effect and a binomial family with a logit link function. Mean infectivity or resistance was then analysed for each time point in a generalized linear mixed model (GLMM) using the lme4 package [26], with time point as a fixed effect and replicate as a random effect. Model coefficients and confidence intervals were transformed from logits to probabilities prior to presentation.

### (g) Time-shift experiment

Because the susceptibility and resistance of bacterial clones to phage from past, present or future time points was determined (table 1), our phenotypic assay also served as a time-shift experiment [27]. Time-shift experiments involve challenging samples of host or pathogen populations from a particular time point against samples of pathogen or host populations from contemporary, past and future host or pathogen populations. Time-shift experiments are a powerful tool to characterize underlying coevolutionary processes and have been used in several host–pathogen systems [28,29], including bacteria–phage [30–32]. Our phage infectivity and host resistance data were analysed as a time-shift experiment by first scoring each pairwise challenge as ‘past’, ‘present’ or ‘future’, with reference to the phage's background compared to the host. Infectivity was then analysed in a GLMM with phage background as a fixed effect and replicate, host time point and phage time point as random effects. Models had a binomial family with a logit link function.

**Table 1.** Pairwise challenges between phage and hosts in the time-shift assay. (Numbers indicate the time points (days post-infection) analysed. Past, present or future refer to if hosts were contemporaneous or not with respect to the phage.)

		phage		
	host	1	4	9
	1	present	future	future
	4	past	present	future
	9	past	past	present

To test for the relative importance of ARD versus FSD, we estimated the strength of the genotype × environment (G × E) effect on infectivity and resistance following Hall *et al.* [31]. Under ARD, all hosts should be more susceptible to phage from their future compared to their past or present, independent of genotype. Environment (E) therefore refers to the time point from which phage originate in pairwise challenges. By contrast, under FSD, different host genotypes will vary in their susceptibility to hosts from their past, present or future. Measuring which proportion of the variation in susceptibility across phage environments can be explained by the interaction between the environment and host genotype (G) can therefore be used to measure the relative contribution of FSD. Increasing values of this proportion (G × E/E) relate to increasing differences in susceptibility among host genotypes. We estimated this by calculating the ratio of the mean square (MS) of an environment-only model to the MS of a G × E model for each replicate at each time point. These ratios were then analysed in a GLMM with a time point as a fixed effect and replicate as a random effect, with a normal family and square root link function.

## 3. Statistical methods

For all experiments, statistical analyses were carried out in R v3.5.0 [33], and graphics were generated using r-base and the ggplot2 package [34]. Model selection followed a nested design, and the final models in all analyses were selected based on the reduction of heteroskedacity, *χ*^2^ tests and Akaike information criterion comparisons [35–37]. Where appropriate, tests were Bonferroni adjusted using the multicomp package [38].

### (a) Spacer sequence analysis

For all bacterial clones that were isolated from the eight replicate experiments where bacteria had been infected with 10^8^ PFU of 2972, expansion of the CRISPR1 and CRISPR3 arrays was analysed using colony polymerase chain reaction (PCR) to determine whether spacer acquisition had taken place (12 clones × 3 time points × 8 replicates = 288 clones in total). The following primers were used: CRISPR1 5′-TGCTGAGACAACCTAGTCTCTC-3′ and 5′-TAAACAGAGCCTCCCTATCC-3′; CRISPR3 5′-CTGAGATTAATAGTGCGATTACG-3′ and 5′-GCTGGATATTCGTATAACATGTC-3′. Clones that had acquired new spacers were further analysed by Sanger sequencing of the amplicon (Source Bioscience, UK), followed by mapping of the spacers against the phage 2972 genome using BLAST followed by manual verification with Geneious v9.1.8 [39]. Spacer diversity was calculated as the pairwise difference (PWD) among nucleotides between spacer sequences. The effect of spacer number and diversity on infectivity was analysed in a GLMM, with either number or diversity as fixed effects and replicate as a random effect. Models had a binomial family and logit link function.

### (b) Phage sequence analysis

To understand whether phages could escape CRISPR immunity through target site (protospacer) mutation, we selected phage clones (out of the 288 total phage clones isolated, see above) and sequenced the protospacer(s) that would match the spacer(s) present among the 12 isolated bacterial clones in a replicate. We then analysed the protospacers and their associated PAMs for single nucleotide polymorphisms (SNPs) that could explain the ability of the phage to overcome CRISPR immunity. For phage clone selection, we used the individual infectivity matrices generated from our phenotypic assays (electronic supplementary material, table S1), and we only sequenced phage clones from infection matrices that showed phage infectivity on hosts that had acquired spacers, i.e. excluded from analysis were phages from those matrices where none of the 12 host clones of a replicate had acquired spacers or where none of the 12 phage clones of a replicate showed infectivity. Sequenced phage clones were taken from 1, 4 and 9 dpi. At least two individual phage clones were selected from each matrix that was analysed, based on their ability to infect CRISPR-immune hosts. When an infection matrix showed a high degree of variation between phage clones, more than two phage clones were analysed so that most of the variation in infectivity would be covered (e.g. in the matrix of replicate 7, T9 phage:T9 host, phage clones 1, 2, 5, 6, 8 and 9 were selected (electronic supplementary material, table S1)). Where possible, one phage clone that did not show infectivity to any of the 12 bacterial clones from each matrix was taken along to serve as a control for protospacer sequencing, alongside an ancestral phage as control. Primers for protospacer sequencing were designed in Geneious by using the available spacer information (electronic supplementary material, tables S2 and S3). PCR amplicons of a total of 51 phage clones were generated by performing PCR on the filtered phage stock, which was followed by Sanger sequencing. To identify point mutations, sequences were first mapped to the 2972 genome using Geneious. SNPs in either the seed sequence or PAM were identified [5,7], and SNP locations were then compared against the protospacer sequence targeted by the CRISPR array of each clone that phage had been challenged against (electronic supplementary material, table S4). Phage with SNP(s) in the seed sequence or PAM of the targeted protospacers were scored as ‘predicted infective’. We found that approximately 70% (241 out of 348) of predicted infectious phage were measured as successfully infecting a host (table 3). The remaining predicted infections that were not measured as successful may be attributable to partial CRISPR resistance or other resistance mechanisms such as surface modification. We also expect some degree of experimental error in our assay given that the detection of lysis zones is a relatively crude method of discerning infectivity/resistance. Using data from the phenotypic assay, we then analysed the effect of the mutation on infectivity. The effect of escape by point mutation on infectivity was modelled in a GLMM as the proportion of infections associated with phage that had an SNP in the protospacer seed sequence or PAM. We analysed the effect of the evolution of the number of SNPs in all targeted sequences matching the host's CRISPR array by first subtracting the number of targeted protospacers that had evolved from the total number of spacers in each host. This gives the number of targeted sequences that had not evolved. The proportion of infections was then modelled against these values. All models included replicate as a random effect, and used a binomial family with a logit link function.

## 4. Results

**Figure 1.**
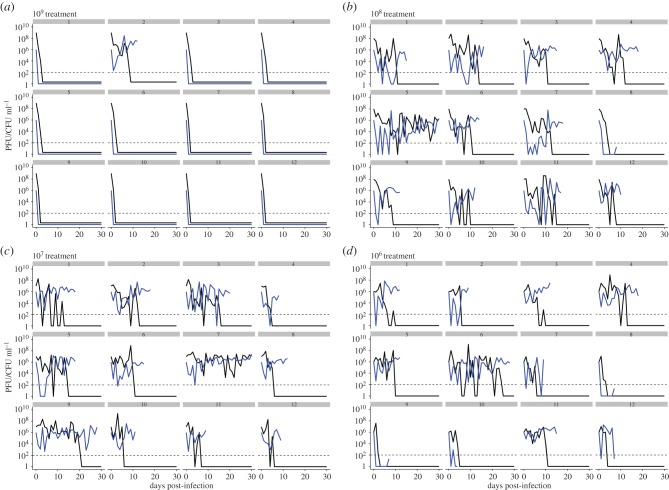
Phage and host population dynamics over time in each replicate. (*a*–*d*) 10^9^–10^6^ PFU phage treatments, respectively, with replicate identity indicated above each sub-panel. Phage titres (plaque-forming units; PFU ml^−1^) are shown in black and host densities (colony-forming units; CFU ml^−1^) are shown in blue. The level of detection is 200 PFU ml^−1^ (dashed line).

We set out to first examine the generality of the previously reported population dynamics following infection of *S. thermophilus* DGCC7710 with a single phage 2972. We therefore infected 12 replicate experimental populations of *S. thermophilus* DGCC7710 with either 10^6^, 10^7^, 10^8^ or 10^9^ PFU of phage 2972 (12 independent replicates per treatment; 48 populations in total) and monitored the bacterial and phage population densities on a daily basis for 30 days. For the first 3 days following infection, phage titres remained fairly constant in most replicates between 10^6^ and 10^8^ PFU ml^−1^, with the exception of the highest phage treatment (10^9^ PFU) where phage and bacteria went extinct in 11 out of 12 replicates (figure 1). Lower phage titres were correlated with higher host densities (*z* = −0.31, 95% confidence interval (CI) = −0.42, −0.19, *p <* 0.0001). With the exception of the 10^9^ treatment, this relationship between phage and host titres was the same among treatments (*F*_2,551_ = 2.24, *p* = 0.11). At 16 days post-infection (dpi), the phage had gone extinct in 44 out of 48 replicates, and phage persisted for the entire 30 day duration of the experiment in two replicates, one each in the 10^7^ and 10^8^ PFU treatments. For the treatments where bacteria survived, the mean time until phage extinction in days was as follows: for the 10^9^ PFU treatment: 2 ± 0.54 days; 10^8^ treatment: 11.50 ± 1.77 days; 10^7^ treatment: 11.50 ± 2.12 days; and 10^6^ treatment: 7.67 ± 1.67 days (mean *±* standard error).

**Figure 2.**
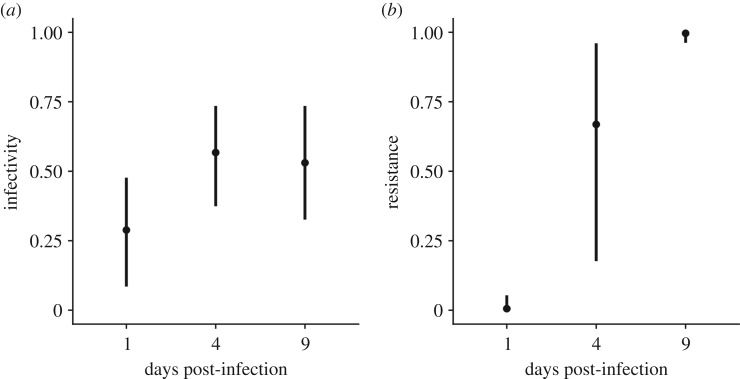
Evolution of infectivity and resistance over time. (*a*) Phage infectivity over time, represented as the proportion of host genotypes from all time points that were infected by a given phage genotype in each replicate. (*b*) Host resistance over time, represented as the proportion of phage genotypes from all time points that were resisted by a host genotype in each replicate. Means and 95% CIs are shown (*n* = 8).

Using these experimental lines, we first determined whether the coexisting bacteria and phage had evolved during their co-culture. Since the population dynamics associated with the 10^6^–10^8^ PFU infection regimes was virtually identical, we decided to limit our downstream analyses to the 10^8^ treatment only. Further, to achieve sufficient power in our analyses and deduce coevolutionary dynamics, we selected replicates where bacteria and phage coexisted for at least 9 days, resulting in a total of eight replicate populations that were examined in detail (figure 1). We then isolated 12 bacterial clones and 12 phage clones from each replicate at 1, 4 and 9 days post-infection (dpi). Using the 288 phage and 288 bacterial isolates, we first examined whether the phage and bacteria had evolved increased infectivity and resistance over time. This was done by measuring the resistance of each individual bacterial clone against all phage clones derived from the same replicate and measuring the infectivity of each individual phage clone against all bacterial clones from the same replicate. This analysis revealed that mean phage infectivity (the proportion of all host genotypes that can be infected by a given phage genotype) increased significantly from 0.29 (CI = 0.08, 0.48) at 1 dpi to 0.57 (CI = 0.37, 0.74) at 4 dpi, but remained stable at 0.53 (CI = 0.33, 0.74) from 4 to 9 dpi. Mean host resistance (the proportion of all phage genotypes resisted by a given host genotype) increased significantly each time point, from 0.01 (CI = 0.00, 0.05) at 1 dpi to 0.67 (CI = 0.18, 0.96) at 4 dpi, and to 0.99 (CI = 0.96, 0.99) at 9 dpi (figure 2). Collectively, these data show that bacteria evolved to resist essentially all phage genotypes by 9 dpi, but phage did not evolve high levels of infectivity to match.

**Figure 3.**
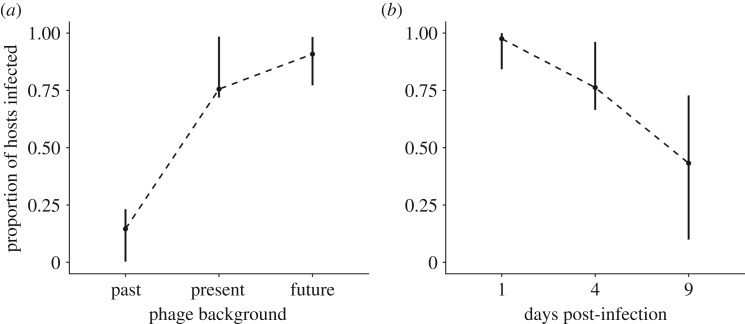
Results from time-shift experiment. (*a*) Proportion of hosts infected when phage were from the host's past, present or future. (*b*) Proportion of hosts infected by phage from the same time point (days post-infection). The dotted line is for illustrative purposes. Means are shown. 95% CIs represent the variation of the mean among replicates (*n* = 100 048).

Having established that bacteria evolved increasing resistance and that phage evolved increasing infectivity over time, we next examined whether both species coevolved; and if so, what type of coevolutionary dynamics were associated with this system. To answer this question, we performed a phenotypic time-shift experiment whereby bacteria were exposed to phage from their past, present and future [27,32] which enabled us to measure infectivity and resistance patterns over time. GLMMs with replicate, host time point and phage time point as random effects and phage background (table 1) as a fixed effect were used to analyse time-shift data. This analysis showed that the original time point of the phage with respect to the host had a significant effect on infectivity ($\chi_{4,10044}^{2}=5.35$, *p* < 0.0001, *R*^2^ = 0.88). Hosts were least susceptible to infection from past phage, more susceptible to contemporaneous phage, and most susceptible to phage from their future (figure 3*a*). This pattern of increasing susceptibility from past to future phage generally held true when each pairwise combination of host and phage time point was considered (electronic supplementary material, figure S1 and table 2). Finally, host susceptibility to phage from the same time point declined consistently from 1 to 9 dpi (figure 3*b*). These data are consistent with an ARD where hosts and pathogens escalate resistance or infectivity over time, but one in which host resistance eventually outpaces pathogen infectivity.

**Table 2.** Mean proportion and 95% confidence interval (CI) of hosts infected and phage resisted in pairwise challenges in the time-shift experiment, broken down by the day from which the host or phage originated. (Values are rounded to two decimal places.)

host	phage	mean infectivity	infectivity 95% CI
t1	t1	0.98	0.80–1.00
t4	t1	0.17	0.01–0.20
t9	t1	0	0.00–0.00
t1	t4	0.90	0.80–1.00
t4	t4	0.74	0.67–0.97
t9	t4	0.01	0.00–0.19
t1	t9	0.85	0.80–0.99
t4	t9	0.79	0.78–0.98
t9	t9	0.36	0.07–0.56

We formally tested for the relative importance of ARD versus FSD in our experiment by estimating the strength of the genotype × environment (G × E) effect on infectivity and resistance [31]. Stronger G × E effects are consistent with stronger FSD (see Experimental methods). This analysis showed that variation among genotypes was weak, consistent with a limited G × E effect (electronic supplementary material, figure S2A,B). The strength of the G × E effect did not change significantly with respect to time point for either phage infectivity ($\chi_{2,24}^{2}=1.93$, *p* = 0.38, *R*^2^ = 0.13) or host resistance ($\chi_{2,24}^{2}=1.46$, *p* = 0.48, *R*^2^ = 0.11). Collectively, these data demonstrate that *S. thermophilus* DGCC7710 and phage 2972 coevolved under these experimental conditions and that the dynamics of their coevolution predominantly follows an arms race.

**Figure 4.**
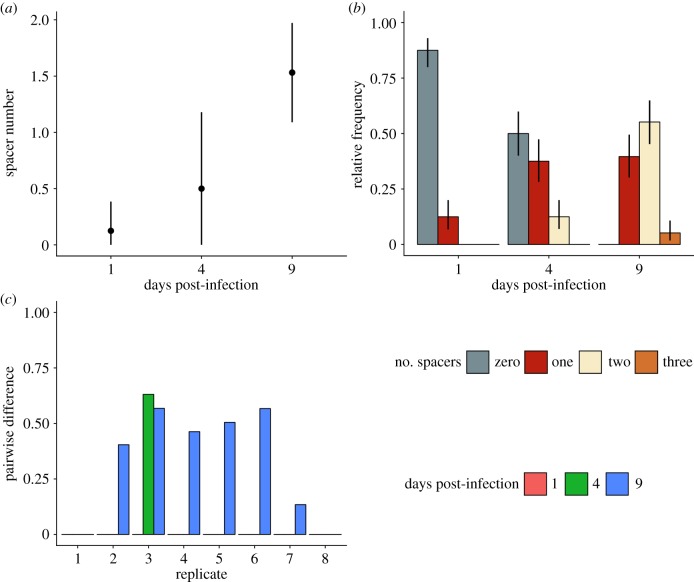
Spacers acquired during coexistence of *S. thermophilus* and phage 2972. (*a*) Number of acquired spacers per clone at each day post-infection (dpi). Means and 95% CIs are shown (*n* = 8). (*b*) Mean relative frequency of clones with different numbers of acquired spacers at each dpi. No clone with greater than three spacers was detected. Means and 95% CIs are shown (*n =* 8). (*c*) Spacer diversity in each replicate, measured as the pairwise differences among spacer sequences in each replicate (*x*-axis) at each time point (colours).

Based on previous studies showing that *S. thermophilus* typically acquires spacers in response to phage exposure [5,15,16], we predicted that this ARD was driven by reciprocal adaptation of the hosts' CRISPR array and the phage protospacers it targets. To test this, we first performed PCR analysis on the CRISPR1 and CRISPR3 loci of each bacterial clone to verify that the mechanistic basis of resistance was in fact because of the acquisition of novel CRISPR spacers. This revealed that the mean number of spacers per clone increased over time ($\chi_{6,1140}^{2}=32.9$, *p* < 0.0001) (figure 4*a*), and that all clones sequenced at 9 dpi had acquired at least one spacer (*M* = 0.55, CI = 0.45, 0.65) (figure 4*b*). Comparison of these spacer sequences with the 2972 genome confirmed that they were acquired from the phage.

**Figure 5.**
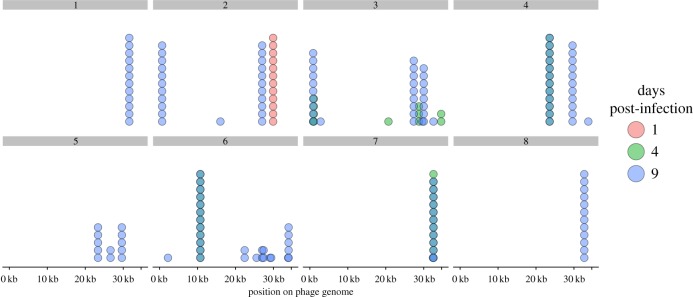
Locations of newly acquired spacers on the phage 2972 genome. Histogram showing the location of acquired spacers in each replicate when mapped against the phage 2972 genome. Each dot represents a clone that had a spacer mapped to that region. Red, green and blue indicate 1, 4 and 9 days post-infection, respectively. Darker colours are the result of the visual overlap between dots. Replicate identity is indicated above each sub-panel.

Using these sequencing data, we determined the level of spacer diversity that naturally evolved within each replicate, because this is an important determinant of CRISPR–phage coevolution [40,41]. Consistent with deep-sequencing analyses of previous co-culture experiments [15,16], our data showed that spacer diversity, measured as the PWD (with 0 indicating all spacers were shared among clones, and 1 indicating all spacers were unique) among spacer sequences, was generally low (grand mean = 0.25). Despite this, there was clear qualitative variation in spacer diversity between different replicates (figure 4*c*). Mean CRISPR genotype richness—the number of different CRISPR alleles we detected—was also low, but increasing, across the sampled time points (1 dpi = 1, 4 dpi = 1.5, 9 dpi = 2.25). The diversity patterns become especially apparent when the spacers are mapped against the phage genome (figure 5) which shows that the spacer composition between time points can change completely, suggestive of selective sweeps of the population.

**Figure 6.**
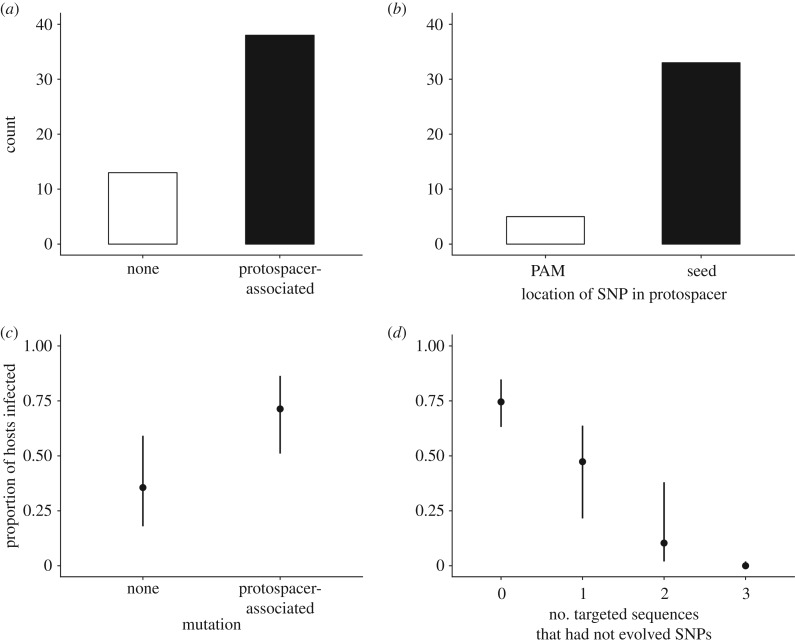
Protospacer sequence analysis and infectivity patterns. (*a*) Histogram showing the number of sequenced phage (out of 56) that did not have a detectable mutation, had a ‘random’ mutation outside of the protospacer or had a ‘protospacer-associated’ mutation either in the protospacer-adjacent motif (PAM) or the seed sequence. (*b*) Number of sequenced phage with protospacer-associated mutations (out of 40) that had a single nucleotide polymorphism (SNP) in either the PAM or seed sequence. (*c*) Mean proportion of hosts infected by phage that did or did not have a protospacer-associated SNP. The effect of random mutations is not included owing to limited sample size. (*d*) Mean proportion of hosts infected by phage that had a full or partial match to the host's CRISPR array in terms of the number of targeted protospacer sequences that had not evolved by point mutation. Means and 95% CIs are shown (*n* = 696).

Consistent with previous theory and data [40,41], we found that host resistance increased with both the number of acquired spacers (*C* = 1.91, *z* = 17.22, *p* < 0.0001, *R*^2^ = 0.66) (electronic supplementary material, figure S3A) and sequence diversity in terms of PWD (*C* = 5.26, *z* = 0.27, *p* < 0.0001, *R*^2^ = 0.44) (electronic supplementary material, figure S3B). These data demonstrate that all clones that had acquired resistance had also acquired at least one novel spacer in either CRISPR1 or CRISPR3, suggesting that resistance is CRISPR-mediated. Further, Sanger sequencing of all CRISPR amplicons confirmed that all spacers which had been acquired indeed targeted the phage 2972 genome (electronic supplementary material, table S2). Spacers most frequently mapped to phage genes encoding hypothetical proteins compared to proteins with known functions, and the targeted genes tended to be at the distal end of the phage genome (electronic supplementary material, table S2). We next tested the hypothesis that the coevolutionary arms race we observed in these experiments was caused by reciprocal adaptation of the phage through the acquisition of point mutations in the target sequences. Such mutations have been observed in a previous co-culture experiment [16] and provide a known mechanism for the phage to overcome CRISPR resistance [5]. To examine whether phage infectivity could be explained by the acquisition of point mutations, we first selected from the phenotypic assays a representative number of 56 different phage clones with different infectivity patterns (i.e. covering both infective and non-infective phenotypes) across the three time points included in the phenotypic assay. We then PCR amplified their protospacer sequences based on the CRISPR spacer sequence data, followed by Sanger sequencing of the amplicons. This showed that 38 out of 51 selected phage clones had acquired at least one SNP in the protospacer sequence or PAM (figure 6*a*); the majority [32] of which were protospacer mutations (figure 6*b*). Further, the majority (approximately 70%) of phage that were predicted to be infectious based on sequence data were able to successfully infect hosts (table 3), indicating that SNPs in the protospacer or PAM were generally sufficient to confer infectivity. The proportion of sequenced phage clones without SNPs in the protospacer or PAM from 4 dpi (10 out of 22) was higher than phage clones from 9 dpi (4 out of 26) (electronic supplementary material, table S3), which is consistent with the idea that CRISPR drives mutation of phage genomes in this empirical system [16]. Crucially, analysis of the infectivity patterns of sequenced phage showed that the mean number of infected hosts was significantly higher when phage had an SNP in the protospacer sequence or PAM compared to phage with no detectable mutations ($\chi_{1,696}^{2}=32.22$, *p* < 0.0001, *R*^2^ = 0.31) (figure 6*c*). Finally, phage that had evolved SNPs in all sequences that were targeted by the host's CRISPR array (0 targeted sequences) had significantly higher infectivity compared to phage that carried one or more unmutated target sequences ($\chi_{1,696}^{2}=59.29$ *p* < 0.0001, *R*^2^ = 0.74) (figure 6*d*). These data demonstrate that the acquisition of point mutations in the protospacer sequence in response to the evolution of CRISPR immunity is the primary mechanism of phage reciprocal adaptation, driving the increase in phage infectivity during the observed ARD.

**Table 3.** Contingency table of pairwise infections that were predicted to lead to phage escape based on protospacer sequence data. (Single nucleotide polymorphisms (SNPs) in either the seed sequence or PAM were identified, and SNP locations were then compared against the protospacer sequence targeted by the CRISPR array of each clone that phage had been challenged against. These were then compared against the pairwise infections measured in the phenotypic assay. ‘+’ indicates a successful infection, ‘−’ indicates no infection.)

		predicted	
		−	+
measured	–	242	107
	+	106	241

## 5. Discussion

*Streptococcus thermophilus* DGCC7710 readily evolves CRISPR-based resistance in response to phage 2972 through spacer acquisition in two active CRISPR loci (CRISPR1 and CRISPR3). In return, phage can escape CRISPR immunity by evolving point mutations in the protospacer sequences targeted by CRISPR. This mechanism of host resistance and pathogen infectivity suggests a possible scenario for coevolution, where bacteria acquire spacers over time and phage accumulate escape mutations.

Consistent with earlier work on *S. thermophilus* and phage 2972 [15–18], we found that, with the exception of the highest initial phage concentration treatment, the phage can coexist with bacteria over many generations despite the presence of CRISPR-based host immunity. Furthermore, our phenotypic data using bacteria and phages isolated during the first 9 dpi demonstrated that they coevolved following an ARD, with hosts and phage evolving increased resistance and infectivity over time, and hosts being more resistant to phage from the past compared to present and future time points. To the best of our knowledge, the evolution of phage resistance is exclusively driven by CRISPR-Cas in this empirical system (i.e. surface modification has not been reported in *S. thermophilus* DGCC7710 in response to phage infections, and we have also never observed it in our experiments). Consistent with this, we found that the underlying mechanism of coexistence during this time span appears to be predominantly reciprocal adaptation of the hosts' CRISPR array and the phage protospacers it targets. Analysis of hosts’ CRISPR arrays shows that they readily acquire phage-derived spacers, that hosts acquire more spacers over time, and that host resistance is strongly associated with both spacer acquisition and spacer number. In turn, phage evolved via point mutations in the targeted protospacers. Correlating this with our phenotypic data shows that such escape phage were on average more infective. Further, we find that phage had evolved SNPs in all target sequences matching the host's CRISPR array were most infective compared to those with an incomplete match.

It is notable that while hosts evolve resistance against essentially all phage, this is not matched by similarly broad phage infectivity range; phage at the last time point (9 dpi) could infect just over half of all hosts. The infectivity of contemporary phage also declines with time, suggesting that the evolution of host resistance ‘outpaces’ that of phage infectivity. This asymmetry between host resistance and phage infectivity is consistent with the idea that bacterial hosts are ‘ahead’ in coevolutionary arms races [42]. While asymmetrical arms races in other studied bacteria–phage systems are generally driven by a binary shift to a phage-resistant surface mutant [8,43], CRISPR–phage interactions suggest an alternative. Hosts can acquire multiple novel spacers with only a marginal cost [44], but phage mutation is limited by mutation supply [43,45,46] (also see Chabas *et al*. in this issue). In addition, full phage infectivity requires mutations in all the protospacers targeted by the host CRISPR array, which becomes increasingly difficult when individual hosts and populations acquire a greater number and diversity of spacer sequences over time [41,45]. It is likely that this asymmetry leads to the repeatable phage extinctions we observed.

Interestingly, this and previous studies occasionally found quasi-stable long-term coexistence of bacteria and phage [15–18]. Previous work suggests that this may be driven by back mutation of resistant hosts towards sensitivity [18]. These two mechanisms for bacteria–phage coexistence may operate in parallel, and their relative importance remains to be investigated. The relative importance of coevolution for phage persistence contrasts with what is observed for *P. aeruginosa* and its phage DMS3vir, where phage are unable to coevolve with the host owing to the high levels of spacer diversity that naturally evolve [41]. In this system, a continuous supply of sensitive hosts can allow for bacteria–phage coexistence [41,46,47].

Our data clearly shows that host genotype diversity richness (i.e. the number of hosts with different CRISPR arrays) increases over time. These data, together with the rapid and repeatable phage extinction after day 9 in our experiment, indirectly support the idea that *S. thermophilus* hosts receive a synergistic benefit from population-level CRISPR diversity in the context of phage infection. Host diversity is a key determinant of pathogen spread (reviewed in [48,49]), and previous work on *P. aeruginosa* PA14 and *S. thermophilus* has shown that population-level CRISPR spacer diversity can limit phage persistence [41]. An understanding that population diversity may provide such a benefit clarifies other results from our data. Although phage infectivity was still possible even with a partial match to the host's CRISPR array, in the context of a mixed, polyclonal host population, such reduced infectivity may limit phage reproduction and transmission, sufficient to cause the rapid extinctions we observed.

In at least some natural environments, bacteria that evolve CRISPR resistance and the phage they target can coexist [3]. This may be owing to CRISPR–phage coevolution, as recently observed for a fish pathogen and its phage [4], but long-term coexistence may also be explained by various other ecological and evolutionary factors that are absent from our simple laboratory environments [50]. For example, previous experiments suggest that longer periods of bacteria–phage coexistence are reached when experimental treatments contained multiple different phages [15,16]. Further, phage under these conditions were found to escape not only by mutation but also by recombination [16]. This is consistent with observations from other natural environments where phage recombinants were correlated with CRISPR activity [3]. These examples highlight how biotic and abiotic complexities may be key in shaping the ecological and evolutionary dynamics of host–pathogen interactions, which we are only starting to understand in the context of CRISPR–phage interactions.

## Supplementary Material

Supplementary Information

## Supplementary Material

Table S1

## Supplementary Material

Table S2

## Supplementary Material

Table S3

## Supplementary Material

Table S4

## Data accessibility

The datasets supporting this article have been uploaded as part of the electronic supplementary material, tables.
