# Supplementary material for: CRISPR-Cas immunity leads to a coevolutionary arms race between Streptococcus thermophilus and lytic phage
Source: Philos Trans R Soc Lond B Biol Sci. 2019 Mar 25;374(1772):20180098. doi: 10.1098/rstb.2018.0098 (PMC6452269; doi:10.1098/rstb.2018.0098)
Supplement: Supplementary Information [file rstb20180098supp1.docx]

| \| 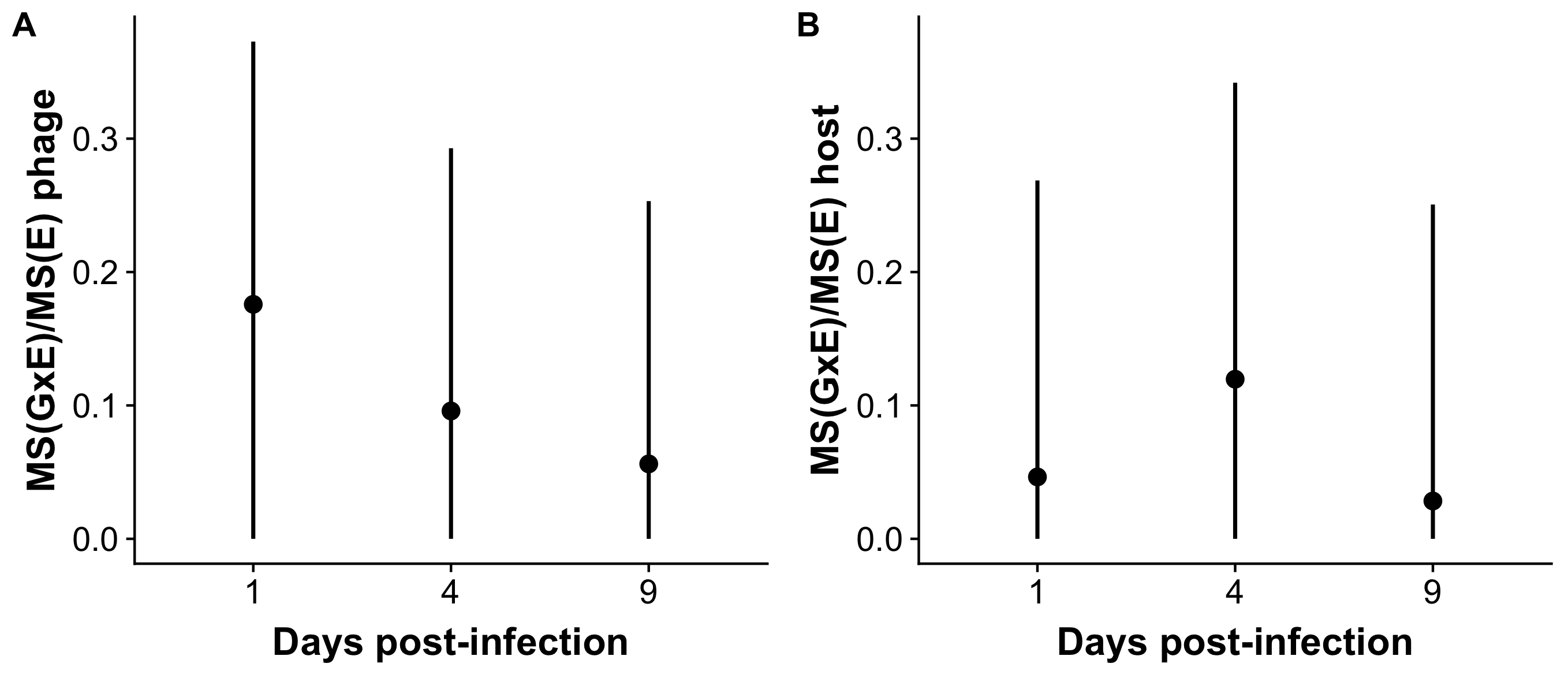 \| \| --- \| \| **Figure S2** Relative importance of fluctuating selection dynamics (FSD) to arms race dynamics (ARD). Under FSD, pathogen genotypes should differ in their infectivity to hosts from contemporary and non-contemporary environments. Scores represent the ratio of the variance in infectivity due to host environment alone explained by variance in infectivity among genotypes, derived from GLMMs with environment as a fixed effect and phage genotype as a random effect (1). Residuals were square-root transformed to introduce a normal distribution in line with model assumptions. Means and 95% CIs are shown are shown (N=8). \| |
| --- | --- | --- |

| **Supplementary Figures**  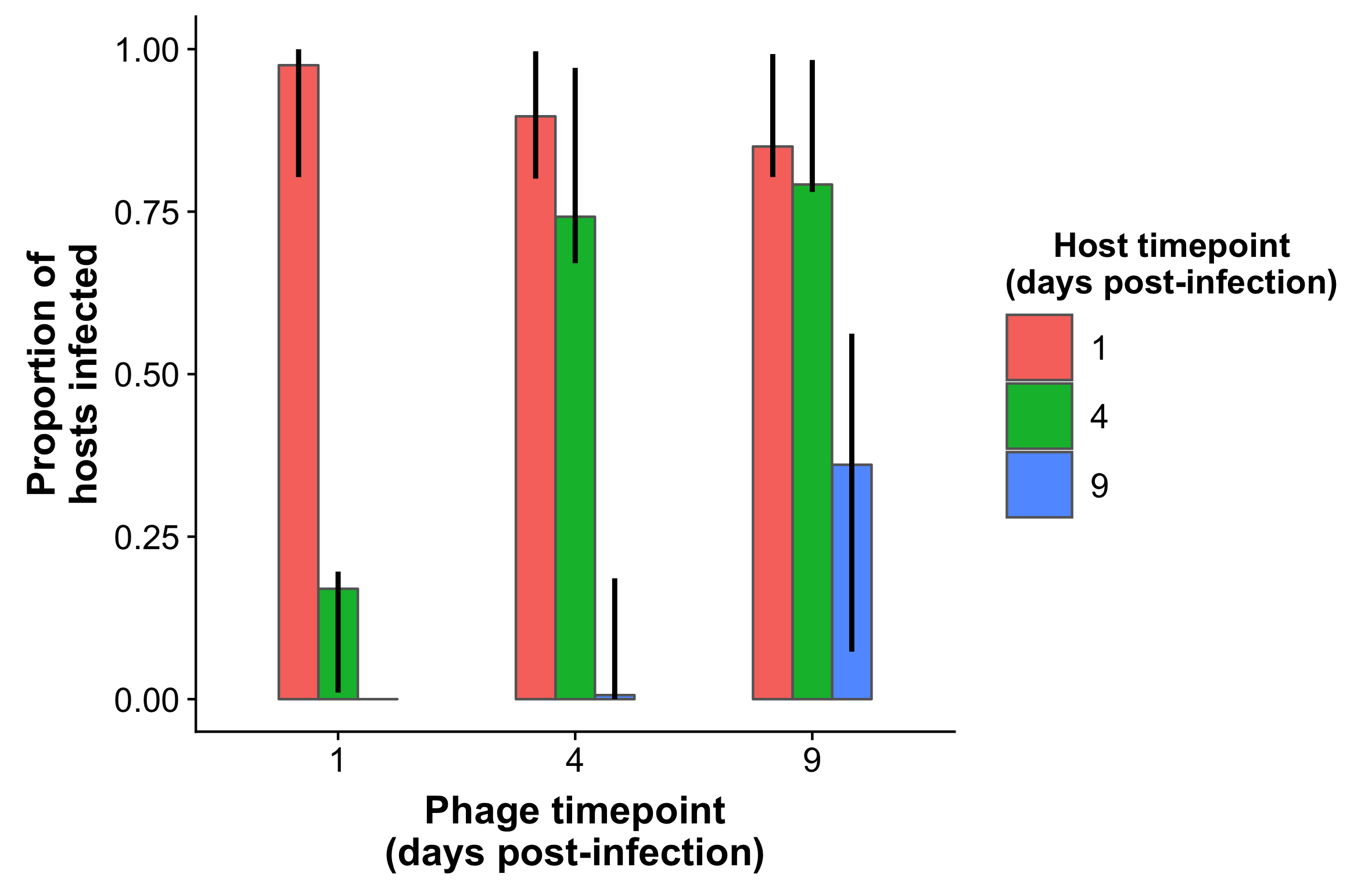 |
| --- |
| **Figure S1** Proportion of hosts resistant to phage that were from the host’s past, present or future from the time-shift experiment. Means and 95% CIs are shown (N=10048). |

| **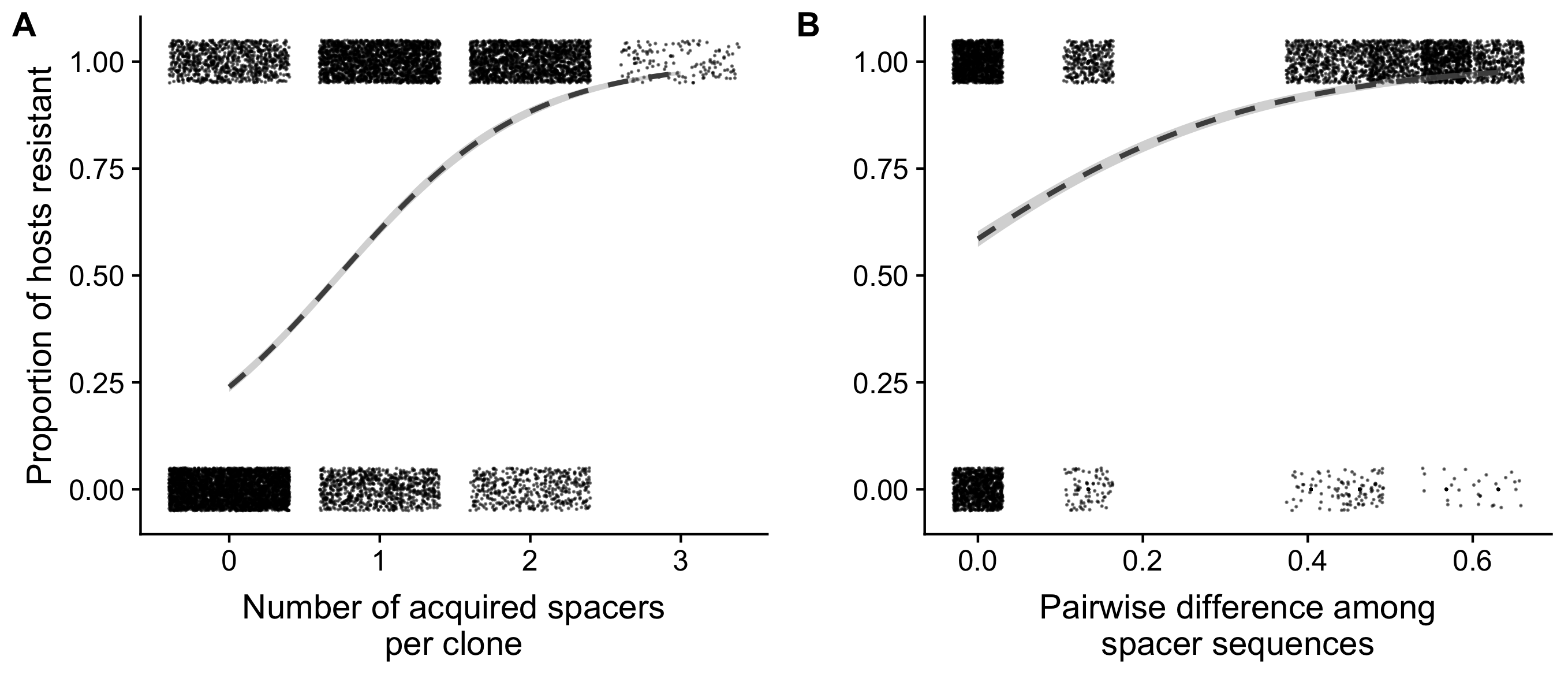** |
| --- |
| **Figure S3** Relationship of host susceptibility to **A**) the number of spacers acquired per clone and **B**) sequence diversity in terms of the pairwise difference among spacer sequences from the phenotypic data. Points show raw data per clone, with random noise added to give a better indication of the number of occurrences of a given measurement. The dashed line is the smoothed logistic (binomial) regression slope fitted through the data. 95% confidence intervals are show in grey. N=10048 |

**Supplementary Table Captions**

**Table S1**

Infectivity matrices from the phenotypic assay (see *Experimental Methods*). The timepoint from which hosts and phage originated is shown. Each 12x12 block is a timeshift challenge within each replicate (1-8). In each block, columns 1-12 are individual bacterial clones and rows 1-12 are individual phage isolates. 1=plaque visible, measured infective; 0=no plaque visible, measured resistant. Phage clones selected for protospacer sequence analysis are indicated by black borders.

**Table S2**

Unique host CRISPR spacers detected by PCR analysis. *Replicate* and *Timepoint* (days post-infection) of the clone(s) which had a given spacer are shown. *Locus* is either CRISPR1 (CR1) or CRISPR3 (CR3). *Start* and *End* are the locations on the phage 2972 genome to which the sequences mapped, given in base pairs. *N* is the number of clones in a replicate X timepoint combination (max. 12) that had the spacer. *GeneID* is the NCBI number of the gene which the spacer mapped against, and refer directly to the. *Gene description* is the function (if known) of the region targeted by the spacer.

**Table S3**

Primer sequences used for protospacer analysis. *Annealing position* indicates where the primer binds to the phage 2972 genome.

**Table S4**

Location(s) of SNP(s) detected by PCR when mapped to the phage 2972 genome. *Replicate* and *Timepoint* (days post-infection) of the phage isolates are shown. *Phage ID* is the number of the phage isolate (rows 1-12 in the infectivity matrices [**Table S1**]). *SNP in protospacer* indicates if the SNP(s) was in the seed sequence or protospacer-adjacent motif of the target protospacer. Locations are given in base pairs.

**Supplementary References**

1. Hall AR, Scanlan PD, Morgan AD, Buckling A. Host–parasite coevolutionary arms races give way to fluctuating selection. Ecology letters. 2011;14(7):635-42.
