# Supplementary material for: CRISPR-Cas immunity leads to a coevolutionary arms race between Streptococcus thermophilus and lytic phage
Source: Philos Trans R Soc Lond B Biol Sci. 2019 Mar 25;374(1772):20180098. doi: 10.1098/rstb.2018.0098 (PMC6452269; doi:10.1098/rstb.2018.0098)
Supplement: Table S1 [file rstb20180098supp2.pdf]

		Hosts (T1)												Hosts (T4)												Hosts (T9)											
		1	2	3	4	5	6	7	8	9	10	11	12	1	2	3	4	5	6	7	8	9	10	11	12	1	2	3	4	5	6	7	8	9	10	11	12
Phage (T1)	1	1	1	1	1	1	1	1	1	1	1	1	1	0	0	1	0	0	0	0	0	0	0	1	0	0	0	0	0	0	0	0	0	0	0	0	0
	2	1	1	1	1	1	1	1	1	1	1	1	1	0	0	1	0	0	0	0	0	0	0	1	0	0	0	0	0	0	0	0	0	0	0	0	0
	3	1	1	1	1	1	1	1	1	1	1	1	1	0	0	1	0	0	0	0	0	0	0	1	0	0	0	0	0	0	0	0	0	0	0	0	0
	4	1	1	1	1	1	1	1	1	1	1	1	1	0	0	1	0	0	0	0	0	0	0	1	0	0	0	0	0	0	0	0	0	0	0	0	0
	5	1	1	1	1	1	1	1	1	1	1	1	1	0	0	1	0	0	0	0	0	0	0	1	0	0	0	0	0	0	0	0	0	0	0	0	0
	6	1	1	1	1	1	1	1	1	1	1	1	1	0	0	1	0	0	0	0	0	0	0	1	0	0	0	0	0	0	0	0	0	0	0	0	0
	7	1	1	1	1	1	1	1	1	1	1	1	1	0	0	1	0	0	0	0	0	0	0	1	0	0	0	0	0	0	0	0	0	0	0	0	0
	8	1	1	1	1	1	1	1	1	1	1	1	1	0	0	1	0	0	0	0	0	0	0	1	0	0	0	0	0	0	0	0	0	0	0	0	0
	9	1	1	1	1	1	1	1	1	1	1	1	1	0	0	1	0	0	0	0	0	0	0	1	0	0	0	0	0	0	0	0	0	0	0	0	0
	10	1	1	1	1	1	1	1	1	1	1	1	1	0	0	1	0	0	0	0	0	0	0	1	0	0	0	0	0	0	0	0	0	0	0	0	0
	11	1	1	1	1	1	1	1	1	1	1	1	1	0	0	1	0	0	0	0	0	0	0	1	0	0	0	0	0	0	0	0	0	0	0	0	0
	12	1	1	1	1	1	1	1	1	1	1	1	1	0	0	1	0	0	0	0	0	0	0	1	0	0	0	0	0	0	0	0	0	0	0	0	0
Phage (T4)	1	1	1	1	1	1	1	1	1	1	1	1	1	0	0	1	0	0	0	0	0	0	0	1	0	0	0	0	0	0	0	0	0	0	0	0	0
	2	1	1	1	1	1	1	1	1	1	1	1	1	0	0	1	0	0	0	0	0	0	1	1	0	0	0	0	0	0	0	0	0	0	0	0	0
	3	1	1	1	1	1	1	1	1	1	1	1	1	0	0	1	0	0	0	0	0	0	0	1	0	0	0	0	0	0	0	0	0	0	0	0	0
	4	1	1	1	1	1	1	1	1	1	1	1	1	0	0	1	0	0	0	0	0	0	0	1	0	0	0	0	0	0	0	0	0	0	0	0	0
	5	1	1	1	1	1	1	1	1	1	1	1	1	0	0	1	0	0	0	0	0	0	0	1	0	0	0	0	0	0	0	0	0	0	0	0	0
	6	1	1	1	1	1	1	1	1	1	1	1	1	0	0	1	0	0	0	0	0	0	0	1	0	0	0	0	0	0	0	0	0	0	0	0	0
	7	1	1	1	1	1	1	1	1	1	1	1	1	1	1	1	1	1	1	1	1	1	0	1	1	0	0	0	0	0	0	0	0	0	0	0	0
	8	1	1	1	1	1	1	1	1	1	1	1	1	0	0	1	0	0	0	0	0	0	0	1	0	0	0	0	0	0	0	0	0	0	0	0	0
	9	1	1	1	1	1	1	1	1	1	1	1	1	0	0	1	0	0	0	0	0	0	1	1	0	0	0	0	0	0	0	0	0	0	0	0	0
	10	1	1	1	1	1	1	1	1	1	1	1	1	0	0	1	0	0	0	0	0	0	1	1	0	0	0	0	0	0	0	0	0	0	0	0	0
	11	1	1	1	1	1	1	1	1	1	1	1	1	0	0	1	0	0	0	0	0	0	0	1	0	0	0	0	0	0	0	0	0	0	0	0	0
	12	1	1	1	1	1	1	1	1	1	1	1	1	0	0	1	0	0	0	0	0	0	0	1	0	0	0	0	0	0	0	0	0	0	0	0	0
Phage (T9)	1	1	1	1	1	1	1	1	1	1	1	1	1	1	1	1	1	1	1	1	1	0	0	1	1	1	1	1	1	1	1	1	0	1	1	1	0
	2	1	1	1	1	1	1	1	1	1	1	1	1	1	1	1	1	1	1	1	1	1	0	1	1	1	1	1	1	1	1	1	0	1	1	1	0
	3	1	1	1	1	1	1	1	1	1	1	1	1	0	0	1	0	0	0	0	0	0	0	1	0	0	0	0	0	0	0	0	0	0	0	0	0
	4	1	1	1	1	1	1	1	1	1	1	1	1	1	1	1	1	1	1	1	1	0	0	1	0	1	1	1	1	1	1	1	0	1	1	1	0
	5	1	1	1	1	1	1	1	1	1	1	1	1	0	0	1	0	0	0	0	0	0	0	1	0	0	0	0	0	1	0	0	0	0	0	0	0
	6	1	1	1	1	1	1	1	1	1	1	1	1	1	1	1	1	1	1	1	1	1	0	1	1	1	1	1	1	1	1	0	1	1	1	1	0
	7	1	1	1	1	1	1	1	1	1	1	1	1	1	1	1	1	1	1	1	1	1	0	1	1	1	1	1	1	1	1	1	1	1	1	1	0
	8	1	1	1	1	1	1	1	1	1	1	1	1	1	1	1	1	1	1	1	1	1	1	1	1	1	1	1	1	1	1	1	1	1	1	1	0
	9	1	1	1	1	1	1	1	1	1	1	1	1	1	1	1	1	1	1	1	1	1	1	1	1	1	1	1	1	1	1	1	1	1	1	1	0
	10	1	1	1	1	1	1	1	1	1	1	1	1	1	1	1	1	1	1	1	1	1	1	1	1	1	1	1	1	1	1	1	1	0	1	1	0
	11	1	1	1	1	1	1	1	1	1	1	1	1	0	0	1	0	0	0	0	0	1	0	1	0	0	0	0	0	1	0	0	1	0	0	0	0
	12	1	1	1	1	1	1	1	1	1	1	1	1	1	1	1	1	1	1	1	1	1	0	1	1	1	1	1	1	1	1	1	1	0	1	1	0

Replicate 1

Phage (T1)	Hosts (T1)												Hosts (T4)												Hosts (T9)											
	1	2	3	4	5	6	7	8	9	10	11	12	1	2	3	4	5	6	7	8	9	10	11	12	1	2	3	4	5	6	7	8	9	10	11	12
1	1	1	1	1	1	1	1	1	1	1	1	1	0	0	0	0	0	0	0	0	0	0	0	0	0	0	0	0	0	0	0	0	0	0	0	0
2	1	1	1	1	1	1	1	1	1	1	1	1	0	0	0	0	0	0	0	0	0	0	0	0	0	0	0	0	0	0	0	0	0	0	0	0
3	1	1	1	1	1	1	1	1	1	1	1	1	0	0	0	0	0	0	0	0	0	0	0	0	0	0	0	0	0	0	0	0	0	0	0	0
4	1	1	1	1	1	1	1	1	1	1	1	1	0	0	0	0	0	0	0	0	0	0	0	0	0	0	0	0	0	0	0	0	0	0	0	0
5	1	0	0	1	1	0	0	0	0	0	1	1	0	0	0	0	0	0	0	0	0	0	0	0	0	0	0	0	0	0	0	0	0	0	0	0
6	1	1	1	1	1	1	1	1	1	1	1	1	0	0	0	0	0	0	0	0	0	0	0	0	0	0	0	0	0	0	0	0	0	0	0	0
7	1	0	1	1	0	0	0	1	0	1	1	0	0	0	0	0	0	0	0	0	0	0	0	0	0	0	0	0	0	0	0	0	0	0	0	0
8	1	0	0	1	0	0	0	1	1	0	1	0	0	0	0	0	0	0	0	0	0	0	0	0	0	0	0	0	0	0	0	0	0	0	0	0
9	1	1	1	1	1	1	1	1	1	0	1	1	0	0	0	0	0	0	0	0	0	0	0	0	0	0	0	0	0	0	0	0	0	0	0	0
10	1	1	1	1	1	1	1	1	1	1	1	1	0	0	0	0	0	0	0	0	0	0	0	0	0	0	0	0	0	0	0	0	0	0	0	0
11	1	1	0	1	0	0	0	1	0	1	0	0	0	0	0	0	0	0	0	0	0	0	0	0	0	0	0	0	0	0	0	0	0	0	0	0
12	1	1	0	1	0	0	0	1	0	1	0	0	0	0	0	0	0	0	0	0	0	0	0	0	0	0	0	0	0	0	0	0	0	0	0	0

Phage (T4)																																				
1	1	1	1	1	1	1	1	1	1	1	1	1	1	1	1	1	1	1	1	1	1	1	1	1	0	0	0	0	0	0	0	0	0	0	0	0
2	1	1	1	1	1	1	1	1	1	1	1	1	1	1	1	1	1	1	1	1	1	1	1	1	0	0	0	0	0	0	0	0	0	0	0	0
3	1	1	1	1	1	1	1	1	1	1	1	1	1	1	1	1	1	1	1	1	1	1	1	1	0	0	0	0	0	0	0	0	0	0	0	0
4	1	1	1	1	1	1	1	1	1	1	1	1	1	1	1	1	1	1	1	1	1	1	1	1	0	1	0	0	0	0	0	0	0	0	0	0
5	1	1	1	1	1	1	1	1	1	1	1	1	1	1	1	1	1	1	1	1	1	1	1	1	0	0	0	0	0	0	0	0	0	1	1	1
6	1	1	1	1	1	1	1	1	1	1	1	1	1	1	1	1	1	1	1	1	1	1	1	1	0	0	0	0	0	0	0	0	0	0	0	0
7	1	1	1	1	1	1	1	1	1	1	1	1	1	1	1	1	1	1	1	1	1	1	1	1	0	0	0	0	0	0	0	0	0	0	0	0
8	1	1	1	1	1	1	1	1	1	1	1	1	1	1	1	1	1	1	1	1	1	1	1	1	0	0	0	0	0	0	0	0	0	0	0	0
9	1	1	1	1	1	1	1	1	1	1	1	1	1	1	1	1	1	1	1	1	1	1	1	1	0	0	0	0	0	0	0	0	0	0	0	0
10	1	1	1	1	1	1	1	1	1	1	1	1	1	1	1	1	1	1	1	1	1	1	1	1	0	0	0	0	0	0	0	0	0	0	0	0
11	1	1	1	1	1	1	1	1	1	1	1	1	1	1	1	0	1	1	0	0	0	0	0	1	0	0	0	0	0	0	0	0	0	0	0	0
12	1	1	1	1	1	1	1	1	1	1	1	1	1	1	1	0	1	1	0	0	0	0	0	1	0	0	0	0	0	0	0	0	0	0	0	0

Phage (T9)																																				
1	0	0	0	0	0	0	0	0	0	0	0	0	1	1	1	1	1	1	1	1	1	1	1	1	1	1	1	1	1	1	1	1	1	1	0	1
2	0	0	0	0	0	0	0	0	0	0	0	0	1	1	1	1	1	1	1	1	1	1	1	1	1	1	1	1	1	0	0	0	0	0	0	0
3	0	0	0	0	0	0	0	0	0	0	0	0	1	1	1	1	1	1	1	1	1	1	1	1	1	0	0	0	0	0	0	0	0	0	0	0
4	0	0	0	0	0	0	0	0	0	0	0	0	1	1	1	1	1	1	1	1	1	1	1	1	1	0	0	0	0	0	0	0	0	0	0	0
5	0	0	0	1	0	0	0	0	0	0	0	0	1	1	1	1	1	1	1	1	1	1	1	1	0	0	0	0	0	0	0	0	0	0	0	0
6	0	0	0	0	0	0	0	0	0	0	0	0	1	1	1	1	1	1	1	1	1	1	1	1	0	0	0	0	0	0	0	0	0	0	0	0
7	0	0	0	0	0	0	0	0	0	0	0	0	1	1	1	1	1	1	1	1	1	1	1	1	0	0	0	0	0	0	0	0	0	0	0	0
8	0	0	0	0	0	0	0	0	0	0	0	0	0	0	0	0	0	0	0	0	0	0	0	0	0	0	0	0	0	0	0	0	0	0	0	0
9	0	0	0	0	0	0	0	0	0	0	0	0	0	0	0	0	0	0	0	0	0	0	0	0	0	0	0	0	0	0	0	0	0	0	0	0
10	0	0	0	0	0	0	0	0	0	0	0	0	0	0	0	0	0	0	0	0	0	0	0	0	0	0	0	0	0	0	0	0	0	0	0	0
11	0	0	0	0	0	0	0	0	0	0	0	0	0	0	1	0	1	1	0	0	0	0	0	0	0	0	0	0	0	1	0	1	1	0	0	0
12	0	0	0	0	0	0	0	0	0	0	0	0	0	0	1	0	1	1	0	0	0	0	0	0	0	0	0	0	0	1	0	1	1	0	0	1

Replicate 2

Hosts (T1)

Phage		1	2	3	4	5	6	7	8	9	10	11	12
Phage (T1)	1	1	1	1	1	1	1	1	1	1	1	1	1
	2	1	1	1	1	1	1	1	1	1	1	1	1
	3	1	1	1	1	1	1	1	1	1	1	1	1
	4	1	1	1	1	1	1	1	1	1	1	1	1
	5	1	1	1	1	1	1	1	1	1	1	1	1
	6	1	1	1	1	1	1	1	1	1	1	1	1
	7	1	1	1	1	1	1	1	1	1	1	1	1
	8	1	1	1	1	1	1	1	1	1	1	1	1
	9	1	1	1	1	1	1	1	1	1	1	1	1
	10	1	1	1	1	1	1	1	1	1	1	1	1
	11	1	1	1	1	1	1	1	1	1	1	1	1
	12	1	1	1	1	1	1	1	1	1	1	1	1
Phage (T4)	1	0	0	0	0	0	0	0	0	0	0	1	0
	2	0	0	0	0	0	0	0	0	0	0	0	0
	3	0	0	0	0	0	1	0	0	0	1	1	0
	4	0	0	1	0	0	0	0	0	0	0	0	0
	5	0	0	0	0	0	0	0	0	0	0	1	0
	6	0	0	0	0	0	0	1	0	0	0	1	1
	7	1	1	1	1	1	1	1	1	1	1	1	1
	8	1	1	1	1	1	1	1	1	1	1	1	0
	9	1	0	0	0	0	0	0	0	0	0	1	0
	10	0	0	0	0	0	1	0	0	0	0	1	0
	11	0	0	1	0	0	0	0	0	0	0	0	0
	12	0	0	0	0	0	0	0	0	0	0	0	0
Phage (T9)	1	1	0	0	0	0	1	0	0	0	0	0	1
	2	1	1	1	1	1	1	1	1	1	1	1	1
	3	1	1	1	1	1	1	1	1	1	1	1	1
	4	0	0	1	0	0	1	0	0	0	0	0	1
	5	1	1	1	1	1	1	1	1	1	1	1	1
	6	1	1	1	1	1	1	1	1	1	1	1	1
	7	1	1	1	1	1	1	1	1	1	1	1	1
	8	1	1	1	1	1	1	1	1	1	1	1	1
	9	1	1	1	1	1	1	1	1	1	1	1	1
	10	1	1	1	1	1	1	1	1	1	1	1	1
	11	1	1	1	1	1	1	1	1	1	1	1	1
	12	1	1	1	1	1	1	1	1	1	1	1	1

Hosts (T4)

Phage		1	2	3	4	5	6	7	8	9	10	11	12
Phage (T1)	1	1	0	0	0	0	0	0	0	0	0	0	0
	2	1	0	0	0	0	0	0	0	0	0	0	0
	3	0	0	0	0	0	0	0	0	0	0	0	0
	4	1	0	0	0	0	0	0	0	0	0	0	0
	5	0	0	0	0	0	0	0	0	0	0	0	0
	6	0	0	0	0	0	0	0	0	0	0	0	0
	7	0	0	0	0	0	0	0	0	0	0	0	0
	8	0	0	0	0	0	0	0	0	0	0	0	0
	9	0	0	0	0	0	0	0	0	0	0	0	0
	10	1	0	0	0	0	0	0	0	0	0	0	0
	11	0	0	0	0	0	0	0	0	0	0	0	0
	12	0	0	0	0	0	0	0	0	0	0	0	0
Phage (T4)	1	0	0	0	0	0	0	0	0	0	0	0	0
	2	0	0	0	0	0	0	0	0	0	0	0	0
	3	0	0	0	0	0	0	0	0	0	0	0	0
	4	0	0	0	0	0	0	0	0	0	0	0	0
	5	0	0	0	0	0	0	0	0	0	0	0	0
	6	0	0	0	0	0	0	0	0	0	0	0	0
	7	0	0	0	0	0	0	0	0	0	0	0	0
	8	0	0	0	0	0	0	0	0	0	0	0	0
	9	0	0	0	0	0	0	0	0	0	0	0	0
	10	0	0	0	0	0	0	0	0	0	0	0	0
	11	0	0	0	0	0	0	0	0	0	0	0	0
	12	0	0	0	0	0	0	0	0	0	0	0	0
Phage (T9)	1	0	0	0	0	0	0	1	0	0	0	0	0
	2	0	0	0	0	0	0	1	0	0	0	0	0
	3	1	0	0	0	0	0	0	0	0	0	0	0
	4	0	0	0	0	0	0	0	0	0	0	0	0
	5	0	0	0	0	0	0	0	0	0	0	0	0
	6	0	0	0	0	0	0	0	0	0	0	0	0
	7	0	0	0	0	0	0	0	0	0	0	0	0
	8	0	0	0	0	0	0	0	0	0	0	0	0
	9	0	0	0	0	0	1	1	0	1	0	0	1
	10	1	0	0	0	0	0	0	0	0	0	0	0
	11	0	0	0	0	0	1	1	0	1	0	0	1
	12	0	0	0	0	0	0	0	0	1	0	0	0

Hosts (T9)

Phage		1	2	3	4	5	6	7	8	9	10	11	12
Phage (T1)	1	0	0	0	0	0	0	0	0	0	0	0	0
	2	0	0	0	0	0	0	0	0	0	0	0	0
	3	0	0	0	0	0	0	0	0	0	0	0	0
	4	0	0	0	0	0	0	0	0	0	0	0	0
	5	0	0	0	0	0	0	0	0	0	0	0	0
	6	0	0	0	0	0	0	0	0	0	0	0	0
	7	0	0	0	0	0	0	0	0	0	0	0	0
	8	0	0	0	0	0	0	0	0	0	0	0	0
	9	0	0	0	0	0	0	0	0	0	0	0	0
	10	0	0	0	0	0	0	0	0	0	0	0	0
	11	0	0	0	0	0	0	0	0	0	0	0	0
	12	0	0	0	0	0	0	0	0	0	0	0	0
Phage (T4)	1	0	0	0	0	0	0	0	0	0	0	0	0
	2	0	0	0	0	0	0	0	0	0	0	0	0
	3	0	0	0	0	0	0	0	0	0	0	0	0
	4	0	0	0	0	0	0	0	0	0	0	0	0
	5	0	0	0	0	0	0	0	0	0	0	0	0
	6	0	0	0	0	0	0	0	0	0	0	0	0
	7	0	0	0	0	0	0	0	0	0	0	0	0
	8	0	0	0	0	0	0	0	0	0	0	0	0
	9	0	0	0	0	0	0	0	0	0	0	0	0
	10	0	0	0	0	0	0	0	0	0	0	0	0
	11	0	0	0	0	0	0	0	0	0	0	0	0
	12	0	0	0	0	0	0	0	0	0	0	0	0
Phage (T9)	1	0	0	0	0	0	0	0	0	0	0	0	0
	2	0	0	0	0	0	1	0	0	0	0	0	0
	3	0	0	0	0	0	0	0	0	0	0	0	0
	4	0	0	0	0	0	0	0	0	0	0	0	0
	5	0	0	0	0	0	0	0	0	0	0	0	0
	6	0	0	0	0	0	0	0	0	0	0	0	0
	7	0	0	0	0	0	0	0	0	0	0	0	0
	8	0	0	0	0	0	0	0	0	0	0	0	0
	9	0	1	1	1	1	1	1	0	1	0	1	1
	10	0	0	0	0	0	0	0	0	0	0	0	0
	11	0	0	0	0	0	1	0	0	0	0	0	0
	12	0	0	0	0	0	0	0	0	0	0	0	0

Replicate 3

		Hosts (T1)											
		1	2	3	4	5	6	7	8	9	10	11	12
Phage (T1)	1	1	0	1	1	1	1	1	1	1	1	1	1
	2	1	0	1	1	1	1	1	1	1	1	1	1
	3	1	0	1	1	1	1	1	1	1	1	1	1
	4	1	0	1	1	1	1	1	1	1	1	1	1
	5	1	0	1	1	1	1	1	1	1	1	1	1
	6	1	0	1	1	1	1	1	1	1	1	1	1
	7	1	0	1	1	1	1	1	1	1	1	1	1
	8	1	0	1	1	1	1	1	1	1	1	1	1
	9	1	0	1	1	1	1	1	1	1	1	1	1
	10	1	0	1	1	1	1	1	1	1	1	1	1
	11	1	0	1	1	1	1	1	1	1	1	1	1
	12	1	0	1	1	1	1	1	1	1	1	1	1
Phage (T4)	1	0	0	0	0	0	0	0	0	0	0	0	0
	2	0	0	0	0	0	0	0	0	0	0	0	0
	3	0	0	0	0	0	0	0	0	0	0	0	0
	4	0	0	0	0	0	0	0	0	0	0	0	0
	5	0	0	0	0	0	1	0	0	0	0	0	0
	6	1	0	0	0	0	0	0	0	0	0	0	0
	7	0	0	0	0	0	0	0	0	0	0	0	0
	8	0	0	0	0	0	0	0	0	0	0	0	0
	9	0	0	0	1	1	1	0	0	0	0	0	0
	10	0	0	0	0	0	0	0	0	0	0	0	0
	11	0	0	0	0	0	0	0	0	0	0	0	0
	12	1	0	1	1	1	1	1	1	1	1	1	1
Phage (T9)	1	0	0	0	0	0	1	0	0	1	0	1	0
	2	1	0	1	0	0	1	1	0	1	0	1	1
	3	1	0	1	0	1	1	0	0	0	0	1	1
	4	0	0	1	0	0	0	1	0	0	0	0	0
	5	1	0	1	0	1	1	1	1	1	1	1	1
	6	0	0	0	1	0	0	0	0	0	0	0	0
	7	0	0	0	1	0	0	0	0	0	0	0	1
	8	1	0	1	0	1	1	1	1	1	1	1	1
	9	0	0	0	0	0	0	0	0	0	0	0	0
	10	0	0	1	1	0	0	0	0	0	0	0	0
	11	0	0	0	1	1	1	1	1	1	1	1	0
	12	0	0	0	0	0	0	0	0	0	0	1	0

		Hosts (T4)											
		1	2	3	4	5	6	7	8	9	10	11	12
Phage (T1)	1	0	0	0	0	0	0	0	0	0	0	0	0
	2	0	0	0	0	0	0	0	0	0	0	0	0
	3	0	0	0	0	0	0	0	0	0	0	0	0
	4	0	0	0	0	0	0	0	0	0	0	0	0
	5	0	0	0	0	0	0	0	0	0	0	0	0
	6	0	0	0	0	0	0	0	0	0	0	0	0
	7	0	0	0	0	0	0	0	0	0	0	0	0
	8	0	0	0	0	0	0	0	0	0	0	0	0
	9	0	0	0	0	0	0	0	0	0	0	0	0
	10	0	0	0	0	0	0	0	0	0	0	0	0
	11	0	0	0	0	0	0	0	0	0	0	0	0
	12	0	0	0	0	0	0	0	0	0	0	0	0
Phage (T4)	1	0	0	0	0	0	0	0	0	0	0	0	0
	2	0	0	0	0	0	0	0	0	0	0	0	0
	3	0	0	0	0	0	0	0	0	0	0	0	0
	4	0	0	0	0	0	0	0	0	0	0	0	0
	5	0	0	0	0	0	0	0	0	0	0	0	0
	6	0	0	0	0	0	0	0	0	0	0	0	0
	7	0	0	0	0	0	0	0	0	0	0	0	0
	8	0	0	0	0	0	0	0	0	0	0	0	0
	9	0	0	0	0	0	0	0	0	0	0	0	0
	10	0	0	0	0	0	0	0	0	0	0	0	0
	11	0	0	0	0	0	0	0	0	0	0	0	0
	12	0	0	0	0	0	0	0	0	0	0	0	0
Phage (T9)	1	1	1	1	1	1	1	1	1	1	1	1	1
	2	0	0	0	0	1	1	0	1	1	0	0	1
	3	0	0	1	0	1	0	0	1	1	0	0	1
	4	0	0	0	0	1	0	0	0	1	0	0	1
	5	0	0	0	0	1	1	0	0	0	1	0	1
	6	0	0	0	0	1	0	0	0	0	0	0	0
	7	1	1	1	1	0	1	1	1	1	1	1	1
	8	1	1	1	1	1	1	1	1	0	0	1	1
	9	1	1	1	1	1	1	1	1	1	0	1	1
	10	1	1	1	1	1	1	1	1	1	1	1	1
	11	1	1	0	0	1	0	0	1	1	1	1	1
	12	1	1	0	1	1	1	1	1	1	1	1	1

		Hosts (T9)											
		1	2	3	4	5	6	7	8	9	10	11	12
Phage (T1)	1	0	0	0	0	0	0	0	0	0	0	0	0
	2	0	0	0	0	0	0	0	0	0	0	0	0
	3	0	0	0	0	0	0	0	0	0	0	0	0
	4	0	0	0	0	0	0	0	0	0	0	0	0
	5	0	0	0	0	0	0	0	0	0	0	0	0
	6	0	0	0	0	0	0	0	0	0	0	0	0
	7	0	0	0	0	0	0	0	0	0	0	0	0
	8	0	0	0	0	0	0	0	0	0	0	0	0
	9	0	0	0	0	0	0	0	0	0	0	0	0
	10	0	0	0	0	0	0	0	0	0	0	0	0
	11	0	0	0	0	0	0	0	0	0	0	0	0
	12	0	0	0	0	0	0	0	0	0	0	0	0
Phage (T4)	1	0	0	0	0	0	0	0	0	0	0	0	0
	2	0	0	0	0	0	0	0	0	0	0	0	0
	3	0	0	0	0	0	0	0	0	0	0	0	0
	4	0	0	0	0	0	0	0	0	0	0	0	0
	5	0	0	0	0	0	0	0	0	0	0	0	0
	6	0	0	0	0	0	0	0	0	0	0	0	0
	7	0	0	0	0	0	0	0	0	0	0	0	0
	8	0	0	0	0	0	0	0	0	0	0	0	0
	9	0	0	0	0	0	0	0	0	0	0	0	0
	10	0	0	0	0	0	0	0	0	0	0	0	0
	11	0	0	0	0	0	0	0	0	0	0	0	0
	12	0	0	0	0	0	0	0	0	0	0	0	0
Phage (T9)	1	1	0	0	1	1	0	1	1	1	1	1	1
	2	1	0	1	1	1	0	1	1	1	1	1	1
	3	1	0	1	1	1	0	1	1	1	1	1	1
	4	0	0	1	0	0	0	0	0	0	0	0	0
	5	0	0	1	1	1	0	1	1	1	1	1	1
	6	0	0	0	0	0	0	0	0	0	0	1	0
	7	1	0	0	0	1	0	1	1	1	1	1	0
	8	0	0	1	1	1	1	1	1	1	1	1	1
	9	0	0	0	1	0	0	1	0	0	0	0	0
	10	0	0	0	0	0	0	1	0	1	1	0	1
	11	0	0	0	1	1	1	1	1	1	1	1	0
	12	0	0	0	0	0	0	0	1	1	1	1	0

Replicate 4

		Hosts (T1)												Hosts (T4)												Hosts (T9)											
		1	2	3	4	5	6	7	8	9	10	11	12	1	2	3	4	5	6	7	8	9	10	11	12	1	2	3	4	5	6	7	8	9	10	11	12
Phage (T1)	1	1	1	1	1	1	1	1	1	1	1	1	0	1	0	0	1	1	0	1	1	1	1	0	1	0	0	0	0	0	0	0	0	0	0	0	0
	2	1	1	1	1	1	1	1	1	1	1	1	1	1	0	0	1	1	0	1	1	1	1	0	0	0	0	0	0	0	0	0	0	0	0	0	0
	3	1	1	1	1	1	1	1	1	1	1	1	1	1	1	1	1	1	1	1	1	1	1	1	1	0	0	0	0	0	0	0	0	0	0	0	0
	4	0	0	0	1	0	0	0	0	1	1	0	0	1	0	0	1	0	0	0	1	1	1	0	0	0	0	0	0	0	0	0	0	0	0	0	0
	5	1	1	1	1	1	1	1	1	1	1	1	1	1	0	0	1	1	0	1	1	1	1	0	1	0	0	0	0	0	0	0	0	0	0	0	0
	6	1	1	1	1	1	1	1	1	1	1	1	1	1	0	0	1	1	1	1	1	1	1	0	1	0	0	0	0	0	0	0	0	0	0	0	0
	7	1	1	1	1	1	1	1	1	1	1	1	1	1	0	0	1	1	0	1	1	1	1	0	1	0	0	0	0	0	0	0	0	0	0	0	0
	8	1	1	1	1	1	1	1	1	1	1	1	1	1	1	1	1	1	1	1	1	1	1	1	1	0	0	0	0	0	0	0	0	0	0	0	0
	9	1	1	1	1	1	1	1	1	1	1	1	1	1	0	0	1	1	0	1	1	1	1	0	1	0	0	0	0	0	0	0	0	0	0	0	0
	10	1	1	1	1	1	1	1	1	1	1	1	1	1	0	0	1	1	0	1	1	1	1	0	1	0	0	0	0	0	0	0	0	0	0	0	0
	11	0	1	1	1	1	1	1	1	1	1	1	0	1	1	0	1	1	0	1	1	1	1	0	0	0	0	0	0	0	0	0	0	0	0	0	0
	12	0	1	1	1	1	1	1	1	1	1	1	1	1	0	0	1	1	0	1	1	1	1	0	1	0	0	0	0	0	0	0	0	0	0	0	0
Phage (T4)	1	1	1	1	1	1	1	1	1	1	1	1	1	1	1	1	1	1	1	1	1	1	1	1	1	0	0	0	0	0	0	0	0	0	0	0	0
	2	1	1	1	1	1	1	1	1	1	1	1	1	1	1	1	1	1	1	1	1	1	1	1	1	0	0	0	0	0	0	0	0	0	0	0	0
	3	1	1	1	1	1	1	1	1	1	1	1	1	1	1	1	1	1	1	1	1	1	1	1	1	0	0	0	0	0	0	0	0	0	0	0	0
	4	1	1	1	1	1	1	1	1	1	1	1	1	1	1	1	1	1	1	1	1	1	1	1	1	0	0	0	0	0	0	0	0	0	0	0	0
	5	1	1	1	1	1	1	1	1	1	1	1	1	1	1	1	1	1	1	1	1	1	1	1	1	0	0	0	0	0	0	0	0	0	0	0	0
	6	1	1	1	1	1	1	1	1	1	1	1	1	1	1	1	1	1	1	1	1	1	1	1	1	0	0	0	0	0	0	0	0	0	0	0	0
	7	1	1	1	1	1	1	1	1	1	1	1	1	1	1	1	1	1	1	1	1	1	1	1	1	0	0	0	0	0	0	0	0	0	0	0	0
	8	1	1	1	1	1	1	1	1	1	1	1	1	1	1	1	1	1	1	1	1	1	1	1	1	0	0	0	0	0	0	0	0	0	0	0	0
	9	1	1	1	1	1	1	1	1	1	1	1	1	1	1	1	1	1	1	1	1	1	1	1	1	0	0	0	0	0	0	0	0	0	0	0	0
	10	1	1	1	1	1	1	1	1	1	1	1	1	1	1	1	1	1	1	1	1	1	1	1	1	0	0	0	0	0	0	0	0	0	0	0	0
	11	1	1	1	1	1	1	1	1	1	1	1	1	1	1	1	1	1	1	1	1	1	1	1	1	0	0	0	0	0	0	0	0	0	0	0	0
	12	1	1	1	1	1	1	1	1	1	1	1	1	1	1	1	1	1	1	1	1	1	1	1	1	0	0	0	0	0	0	0	0	0	0	0	0
Phage (T9)	1	1	1	1	1	1	1	1	1	1	1	1	1	1	1	1	1	1	1	1	1	1	1	1	1	0	0	0	0	0	0	0	0	0	0	0	0
	2	1	1	1	1	1	1	1	1	1	1	1	1	1	1	1	1	1	1	1	1	1	1	1	1	0	0	0	0	0	0	0	0	0	0	0	0
	3	1	1	1	1	1	1	1	1	1	1	1	1	1	1	1	1	1	1	1	1	1	1	1	1	0	0	0	0	0	0	0	0	0	0	0	0
	4	1	1	1	1	1	1	1	1	1	1	1	1	1	1	1	1	1	1	1	1	1	1	1	1	0	0	0	0	0	0	0	0	0	0	0	0
	5	1	1	1	1	1	1	1	1	1	1	1	1	1	1	1	1	1	1	1	1	1	1	1	1	0	0	0	0	0	0	0	0	0	0	0	0
	6	1	1	1	1	1	1	1	1	1	1	1	1	1	1	1	1	1	1	1	1	1	1	1	1	0	0	0	0	0	0	0	0	0	0	0	0
	7	1	1	1	1	1	1	1	1	1	1	1	1	1	1	1	1	1	1	1	1	1	1	1	1	0	0	0	0	0	0	0	0	0	0	0	0
	8	1	1	1	1	1	1	1	1	1	1	1	1	1	1	1	1	1	1	1	1	1	1	1	1	0	0	0	0	0	0	0	0	0	0	0	0
	9	1	1	1	1	1	1	1	1	1	1	1	1	1	1	1	1	1	1	1	1	1	1	1	1	0	0	0	0	0	0	0	0	0	0	0	0
	10	1	1	1	1	1	1	1	1	1	1	1	1	1	1	1	1	1	1	1	1	1	1	1	1	0	0	0	0	0	0	0	0	0	0	0	0
	11	1	1	1	1	1	1	1	1	1	1	1	1	1	1	1	1	1	1	1	1	1	1	1	1	0	0	0	0	0	0	0	0	0	0	0	0
	12	1	1	1	1	1	1	1	1	1	1	1	1	1	1	1	1	1	1	1	1	1	1	1	1	0	0	0	0	0	0	0	0	0	0	0	0

Replicate 5

		Hosts (T1)											
		1	2	3	4	5	6	7	8	9	10	11	12
Phage (T1)	1	1	1	1	1	1	1	1	1	1	1	1	1
	2	1	1	1	1	1	1	1	1	1	1	1	1
	3	1	1	1	1	1	1	1	1	1	1	1	1
	4	1	1	1	1	1	1	1	1	1	1	1	1
	5	1	1	1	1	1	1	1	1	1	1	1	1
	6	1	1	1	1	1	1	1	1	1	1	1	1
	7	1	1	1	1	1	1	1	1	1	1	1	1
	8	1	1	1	1	1	1	1	1	1	1	1	1
	9	1	1	1	1	1	1	1	1	1	1	1	1
	10	1	1	1	1	1	1	1	1	1	1	1	1
	11	1	1	1	1	1	1	1	1	1	1	1	1
	12	1	1	1	1	1	1	1	1	1	1	1	1
Phage (T4)	1	1	1	1	1	1	1	1	1	1	1	1	1
	2	1	1	1	1	1	1	1	1	1	1	1	1
	3	1	1	1	1	1	1	1	1	1	1	1	1
	4	1	1	1	1	1	1	1	1	1	1	1	1
	5	1	1	1	1	1	1	1	1	1	1	1	1
	6	1	1	1	1	1	1	1	1	1	1	1	1
	7	1	1	1	1	1	1	1	1	1	1	1	1
	8	1	1	1	1	1	1	1	1	1	1	1	1
	9	1	1	1	1	1	1	1	1	1	1	1	1
	10	1	1	1	1	1	1	1	1	1	1	1	1
	11	1	1	1	1	1	1	1	1	1	1	1	1
	12	1	1	1	1	1	1	1	1	1	1	1	1
Phage (T9)	1	0	0	1	1	1	1	1	1	1	1	1	1
	2	0	1	1	1	1	1	1	1	1	1	1	0
	3	1	0	1	1	1	1	1	1	1	1	1	1
	4	0	1	1	1	1	1	1	1	1	1	1	0
	5	0	1	1	1	1	1	1	1	1	1	1	1
	6	0	1	0	0	1	1	1	1	1	1	1	0
	7	1	0	0	0	1	1	1	1	1	1	0	1
	8	0	0	0	0	0	0	0	0	0	0	0	0
	9	1	1	1	1	1	1	1	1	1	1	1	1
	10	1	0	0	1	1	1	1	1	1	1	1	0
	11	1	0	0	0	0	0	0	0	1	0	1	0
	12	0	0	1	0	0	0	0	0	0	0	1	0

		Hosts (T4)											
		1	2	3	4	5	6	7	8	9	10	11	12
Phage (T1)	1	0	0	0	0	0	0	0	0	0	0	0	0
	2	0	0	0	0	0	0	0	0	0	0	0	0
	3	0	0	0	0	0	0	0	0	0	0	0	0
	4	0	0	0	0	0	0	0	0	0	0	0	0
	5	0	0	1	0	0	0	0	0	0	0	0	0
	6	0	0	0	0	0	0	0	0	0	0	0	0
	7	0	0	0	0	0	0	0	0	0	0	0	0
	8	0	0	0	0	0	0	0	0	0	0	0	0
	9	0	0	1	0	0	0	0	0	0	0	0	0
	10	0	0	1	0	0	0	0	0	0	0	0	0
	11	0	0	1	0	0	0	0	0	0	0	0	0
	12	0	0	0	0	0	0	0	0	0	0	0	0
Phage (T4)	1	1	1	1	1	1	1	1	1	1	1	1	1
	2	1	1	1	1	1	1	1	1	1	1	1	1
	3	1	1	1	1	1	1	1	1	1	1	1	1
	4	1	1	1	1	0	1	1	1	1	1	1	1
	5	1	1	1	1	1	1	1	1	0	1	1	1
	6	1	1	1	1	1	1	1	1	0	1	1	1
	7	1	1	1	1	1	1	1	1	1	1	1	1
	8	1	1	1	1	1	1	1	1	1	1	1	1
	9	1	1	1	1	1	1	1	1	1	0	1	1
	10	1	1	1	1	1	1	1	1	1	1	1	1
	11	1	1	1	1	1	1	1	1	1	1	1	1
	12	1	1	1	1	1	1	1	1	1	1	1	1
Phage (T9)	1	0	1	1	1	0	0	1	1	1	1	1	0
	2	0	0	1	1	0	0	1	1	1	0	1	0
	3	0	0	0	1	0	0	1	0	0	0	0	0
	4	0	1	0	1	1	0	0	0	0	0	0	0
	5	0	1	1	1	0	0	1	0	0	0	0	1
	6	0	1	1	1	1	0	1	0	0	0	0	1
	7	0	1	0	1	1	0	0	0	0	0	0	0
	8	0	0	0	0	0	0	0	0	0	0	0	0
	9	1	1	1	1	1	1	1	1	1	1	1	1
	10	0	1	1	1	1	1	1	0	0	0	1	0
	11	0	0	0	0	0	1	1	0	0	0	0	0
	12	0	0	1	0	0	0	1	0	0	0	0	0

		Hosts (T9)											
		1	2	3	4	5	6	7	8	9	10	11	12
Phage (T1)	1	0	0	0	0	0	0	0	0	0	0	0	0
	2	0	0	0	0	0	0	0	0	0	0	0	0
	3	0	0	0	0	0	0	0	0	0	0	0	0
	4	0	0	0	0	0	0	0	0	0	0	0	0
	5	0	0	0	0	0	0	0	0	0	0	0	0
	6	0	0	0	0	0	0	0	0	0	0	0	0
	7	0	0	0	0	0	0	0	0	0	0	0	0
	8	0	0	0	0	0	0	0	0	0	0	0	0
	9	0	0	0	0	0	0	0	0	0	0	0	0
	10	0	0	0	0	0	0	0	0	0	0	0	0
	11	0	0	0	0	0	0	0	0	0	0	0	0
	12	0	0	0	0	0	0	0	0	0	0	0	0
Phage (T4)	1	0	0	0	0	0	0	0	0	0	0	0	0
	2	0	0	0	0	0	0	0	0	0	0	0	0
	3	0	0	0	0	0	0	0	0	0	0	0	0
	4	0	0	0	0	0	0	0	0	0	0	0	0
	5	0	0	0	0	0	0	0	0	0	0	0	0
	6	0	0	0	0	0	0	0	0	0	0	0	0
	7	0	0	0	0	0	0	0	0	0	0	0	0
	8	0	0	0	0	0	0	0	0	0	0	0	0
	9	0	0	0	0	0	0	0	0	0	0	0	0
	10	0	0	0	0	0	0	0	0	0	0	0	0
	11	0	0	0	0	0	0	0	0	0	0	0	0
	12	0	0	0	0	0	0	0	0	0	0	0	0
Phage (T9)	1	0	0	0	0	0	0	0	0	0	0	0	0
	2	0	0	0	0	0	0	0	0	0	0	0	0
	3	0	0	0	0	0	0	0	0	0	0	0	0
	4	0	0	0	0	0	0	0	0	0	0	0	0
	5	0	0	0	0	0	0	0	0	0	0	0	0
	6	0	0	0	0	0	0	0	0	0	0	0	0
	7	0	0	0	0	0	0	0	0	0	0	0	0
	8	0	0	0	0	0	0	0	0	0	0	0	0
	9	0	0	0	0	0	0	0	0	0	0	0	0
	10	0	0	0	0	0	0	0	0	0	0	0	0
	11	0	0	0	0	0	0	0	0	0	0	0	0
	12	0	0	0	0	0	0	0	0	0	0	0	0

Replicate 6

		Hosts (T1)											
		1	2	3	4	5	6	7	8	9	10	11	12
Phage (T1)	1	1	1	1	1	1	1	1	1	1	1	1	1
	2	1	1	1	1	1	1	0	0	0	1	0	1
	3	1	1	0	1	1	1	1	1	1	1	1	1
	4	1	1	1	1	1	1	1	1	1	1	1	1
	5	1	1	1	1	1	1	1	1	1	1	1	1
	6	1	1	1	1	1	1	1	1	1	1	1	1
	7	1	1	1	1	1	1	1	1	1	1	1	1
	8	1	1	1	1	1	1	1	1	1	1	1	1
	9	1	1	1	1	1	1	1	1	1	1	1	1
	10	1	1	1	1	1	1	1	1	1	1	1	1
	11	1	0	0	1	0	0	0	0	0	0	1	0
	12	1	1	1	1	1	1	1	1	1	0	1	1
Phage (T4)	1	1	1	1	1	1	1	1	1	1	1	1	1
	2	1	1	1	1	1	1	1	1	1	1	1	1
	3	1	1	1	1	1	1	1	1	1	1	1	1
	4	1	1	1	1	1	1	1	1	1	1	1	1
	5	1	1	1	1	1	1	1	1	1	1	1	1
	6	1	1	1	1	1	1	1	1	1	1	1	1
	7	1	1	1	1	1	1	1	1	1	1	1	1
	8	1	1	1	1	1	1	1	1	1	1	1	1
	9	1	1	1	1	1	1	1	1	1	1	1	1
	10	1	1	1	1	1	1	1	1	1	1	1	1
	11	1	1	1	1	1	1	1	1	1	1	1	1
	12	1	1	1	1	1	1	1	1	1	1	1	1
Phage (T9)	1	1	1	1	1	1	1	1	1	1	1	1	1
	2	1	1	1	1	1	1	1	1	1	1	1	1
	3	1	1	1	1	1	1	1	1	1	1	1	1
	4	1	1	1	1	1	1	1	1	1	1	1	1
	5	1	1	1	1	1	1	1	1	1	1	1	1
	6	1	1	1	1	1	1	1	1	1	1	1	1
	7	1	1	1	1	1	1	1	1	1	1	1	1
	8	1	1	1	1	1	1	1	1	1	1	1	1
	9	1	1	1	1	1	1	1	1	1	1	1	1
	10	1	1	1	1	1	1	1	1	1	1	1	1
	11	1	1	1	1	1	1	1	1	1	1	1	1
	12	1	1	1	1	1	1	1	1	1	1	1	1

		Hosts (T4)											
		1	2	3	4	5	6	7	8	9	10	11	12
Phage (T1)	1	0	0	0	0	0	0	0	0	0	0	0	0
	2	0	0	0	0	0	0	0	0	0	0	0	0
	3	0	0	0	0	0	0	0	0	0	0	0	0
	4	0	0	0	0	0	0	0	0	0	0	0	0
	5	0	0	0	0	0	0	0	0	0	0	0	0
	6	0	0	0	0	0	0	0	0	0	0	0	0
	7	0	0	0	0	0	0	0	0	0	0	0	0
	8	0	0	0	0	0	0	0	0	0	0	0	0
	9	0	0	0	0	0	0	0	0	0	0	0	0
	10	0	0	0	0	0	0	0	0	0	0	0	0
	11	0	0	0	0	0	0	0	0	0	0	0	0
	12	0	0	0	0	0	0	0	0	0	0	0	0
Phage (T4)	1	1	1	1	1	1	1	1	1	1	0	1	1
	2	1	1	1	1	1	1	1	1	1	0	1	1
	3	1	1	1	1	1	1	1	1	1	0	1	1
	4	1	1	1	1	1	1	1	1	1	0	1	1
	5	1	1	1	1	1	1	1	1	1	0	1	1
	6	1	1	1	1	1	1	1	1	1	0	1	1
	7	1	1	1	1	1	1	1	1	1	0	1	1
	8	1	1	1	1	1	1	1	1	1	0	1	1
	9	1	1	1	1	1	1	1	1	1	0	1	1
	10	1	1	1	1	1	1	1	1	1	0	1	1
	11	1	1	1	1	1	1	1	1	1	0	1	1
	12	1	1	1	1	0	1	0	1	1	0	1	1
Phage (T9)	1	1	1	1	1	1	1	1	1	1	0	1	1
	2	1	1	1	1	1	1	1	1	1	0	1	1
	3	1	1	1	1	1	1	1	1	1	0	1	1
	4	1	1	1	1	1	1	1	1	1	0	1	1
	5	1	1	1	1	1	1	1	1	1	0	1	1
	6	1	1	1	1	1	1	1	1	1	0	1	1
	7	1	1	1	1	1	1	1	1	1	0	1	1
	8	1	1	1	1	1	1	1	1	1	0	1	1
	9	1	1	1	1	1	1	1	1	1	0	1	1
	10	1	1	1	1	1	1	1	1	1	0	1	1
	11	1	1	1	1	1	1	1	1	1	0	1	1
	12	1	1	1	1	1	1	1	1	1	0	1	1

		Hosts (T9)											
		1	2	3	4	5	6	7	8	9	10	11	12
Phage (T1)	1	0	0	0	0	0	0	0	0	0	0	0	0
	2	0	0	0	0	0	0	0	0	0	0	0	0
	3	0	0	0	0	0	0	0	0	0	0	0	0
	4	0	0	0	0	0	0	0	0	0	0	0	0
	5	0	0	0	0	0	0	0	0	0	0	0	0
	6	0	0	0	0	0	0	0	0	0	0	0	0
	7	0	0	0	0	0	0	0	0	0	0	0	0
	8	0	0	0	0	0	0	0	0	0	0	0	0
	9	0	0	0	0	0	0	0	0	0	0	0	0
	10	0	0	0	0	0	0	0	0	0	0	0	0
	11	0	0	0	0	0	0	0	0	0	0	0	0
	12	0	0	0	0	0	0	0	0	0	0	0	0
Phage (T4)	1	0	0	0	0	0	0	0	0	0	1	0	0
	2	0	0	0	0	0	0	0	0	0	1	0	0
	3	0	0	0	0	0	0	0	0	0	1	0	0
	4	0	0	0	0	0	0	0	0	0	1	0	0
	5	0	0	0	0	0	0	0	0	0	1	0	0
	6	0	0	0	0	0	0	0	0	0	1	0	0
	7	0	0	0	0	0	0	0	0	0	1	0	0
	8	0	0	0	0	0	0	0	0	0	1	0	0
	9	0	0	0	0	0	0	0	0	0	1	0	0
	10	0	0	0	0	0	0	0	0	0	1	0	0
	11	0	0	0	0	0	0	0	0	0	1	0	0
	12	0	0	0	0	0	0	0	0	0	1	0	0
Phage (T9)	1	1	0	1	1	0	1	1	1	1	1	1	1
	2	0	0	1	1	0	1	1	1	1	1	1	1
	3	1	0	1	0	0	0	0	0	1	1	0	0
	4	1	0	1	1	0	1	0	0	1	1	1	1
	5	1	0	1	1	0	1	1	0	1	1	1	1
	6	1	0	1	1	0	1	1	0	1	1	1	1
	7	1	0	1	1	0	1	0	0	1	1	1	1
	8	1	0	1	1	0	1	0	0	1	1	1	1
	9	0	0	0	0	0	0	0	0	0	1	0	0
	10	0	0	0	0	0	0	0	0	0	1	0	0
	11	0	0	0	0	0	0	0	0	0	1	0	0
	12	0	0	1	0	0	0	0	0	1	1	0	0

Replicate 7

		Hosts (T1)												Hosts (T4)												Hosts (T9)											
		1	2	3	4	5	6	7	8	9	10	11	12	1	2	3	4	5	6	7	8	9	10	11	12	1	2	3	4	5	6	7	8	9	10	11	12
Phage (T1)	1	1	1	1	1	1	1	1	1	1	1	1	1	1	1	1	1	1	1	1	1	1	1	1	1	0	0	0	0	0	0	0	0	0	0	0	0
	2	1	1	1	1	1	1	1	1	1	1	1	1	1	1	1	1	1	1	1	1	1	1	1	1	0	0	0	0	0	0	0	0	0	0	0	0
	3	1	1	1	1	1	1	1	1	1	1	1	1	1	1	1	1	1	1	1	1	1	1	1	1	0	0	0	0	0	0	0	0	0	0	0	0
	4	1	1	1	1	1	1	1	1	1	1	1	1	1	1	1	1	1	1	1	1	1	1	1	1	0	0	0	0	0	0	0	0	0	0	0	0
	5	1	1	1	1	1	1	1	1	1	1	1	1	1	1	1	1	1	1	1	1	1	1	1	1	0	0	0	0	0	0	0	0	0	0	0	0
	6	1	1	1	1	1	1	1	1	1	1	1	1	1	1	1	1	1	1	1	1	1	1	1	1	0	0	0	0	0	0	0	0	0	0	0	0
	7	1	1	1	1	1	1	1	1	1	1	1	1	1	1	1	1	1	1	1	1	1	1	1	1	0	0	0	0	0	0	0	0	0	0	0	0
	8	1	1	1	1	1	1	1	1	1	1	1	1	1	1	1	1	1	1	1	1	1	1	1	1	0	0	0	0	0	0	0	0	0	0	0	0
	9	1	1	1	1	1	1	1	1	1	1	1	1	1	1	1	1	1	1	1	1	1	1	1	1	0	0	0	0	0	0	0	0	0	0	0	0
	10	1	1	1	1	1	1	1	1	1	1	1	1	1	1	1	1	1	1	1	1	1	1	1	1	0	0	0	0	0	0	0	0	0	0	0	0
	11	1	1	1	1	1	1	1	1	1	1	1	1	1	1	1	1	1	1	1	1	1	1	1	1	0	0	0	0	0	0	0	0	0	0	0	0
	12	1	1	1	1	1	1	1	1	1	1	1	1	1	1	1	1	1	1	1	1	1	1	1	1	0	0	0	0	0	0	0	0	0	0	0	0
Phage (T4)	1	1	1	1	1	1	1	1	1	1	1	1	1	1	1	1	1	1	1	1	1	1	1	1	1	0	0	1	1	1	1	1	0	1	1	1	1
	2	1	1	1	1	1	1	1	1	1	1	1	1	1	1	1	1	1	1	1	1	1	1	1	1	0	0	0	1	0	0	0	0	0	1	0	1
	3	1	1	1	1	1	1	1	1	1	1	1	1	1	1	1	1	1	1	1	1	1	1	1	1	0	0	0	0	0	0	1	0	0	0	0	0
	4	1	1	1	1	1	1	1	1	1	1	1	1	1	1	1	1	1	1	1	1	1	1	1	1	0	0	0	0	0	0	0	0	0	0	1	1
	5	1	1	1	1	1	1	1	1	1	1	1	1	1	1	1	1	1	1	1	1	1	1	1	1	0	0	0	0	1	0	1	0	0	1	0	1
	6	1	1	1	1	1	1	1	1	1	1	1	1	1	1	1	1	1	1	1	1	1	1	1	1	0	0	1	1	1	1	1	0	0	0	1	1
	7	1	1	1	1	1	1	1	1	1	1	1	1	1	1	1	1	1	1	1	1	1	1	1	1	1	0	1	0	0	0	0	0	0	0	1	0
	8	1	1	1	1	1	1	1	1	1	1	1	1	1	1	1	1	1	1	1	1	1	1	1	1	0	0	0	0	0	0	0	0	0	0	0	0
	9	1	1	1	1	1	1	1	1	1	1	1	1	1	1	1	1	1	1	1	1	1	1	1	1	0	0	0	0	0	1	0	0	0	0	0	1
	10	1	1	1	1	1	1	1	1	1	1	1	1	1	1	1	1	1	1	1	1	1	1	1	1	1	0	0	1	0	0	1	0	0	1	0	1
	11	1	1	1	1	1	1	1	1	1	1	1	1	1	1	1	1	1	1	1	1	1	1	1	1	1	0	0	0	0	0	0	0	0	0	0	1
	12	1	1	1	1	1	1	1	1	1	1	1	1	1	1	1	1	1	1	1	1	1	1	1	1	1	0	0	1	0	0	1	0	0	1	0	1
Phage (T9)	1	1	1	1	1	1	1	1	1	1	1	1	1	1	1	1	1	1	1	1	1	1	1	1	1	1	1	1	1	1	1	1	1	1	1	1	1
	2	1	1	1	1	1	1	1	1	1	1	1	1	1	1	1	1	1	1	1	1	1	1	1	1	1	1	1	1	1	1	1	1	1	1	1	1
	3	1	1	1	1	1	1	1	1	1	1	1	1	1	1	1	1	1	1	1	1	1	1	1	1	1	1	1	1	1	1	1	1	1	1	1	1
	4	1	1	1	1	1	1	1	1	1	1	1	1	1	1	1	1	1	1	1	1	1	1	1	1	1	1	1	1	1	1	1	1	1	1	1	1
	5	1	1	1	1	1	1	1	1	1	1	1	1	1	1	1	1	1	1	1	1	1	1	1	1	1	1	1	1	1	1	1	1	1	1	1	1
	6	1	1	1	1	1	1	1	1	1	1	1	1	1	1	1	1	1	1	1	1	1	1	1	1	1	1	1	1	1	1	1	1	1	1	1	1
	7	1	1	1	1	1	1	1	1	1	1	1	1	1	1	1	1	1	1	1	1	1	1	1	1	1	1	1	1	1	1	1	1	1	1	1	1
	8	1	1	1	1	1	1	1	1	1	1	1	1	1	1	1	1	1	1	1	1	1	1	1	1	1	1	1	1	1	1	1	1	1	1	1	1
	9	1	1	1	1	1	1	1	1	1	1	1	1	1	1	1	1	1	1	1	1	1	1	1	1	1	1	1	1	1	1	1	1	1	1	1	1
	10	1	1	1	1	1	1	1	1	1	1	1	1	1	1	1	1	1	1	1	1	1	1	1	1	1	1	1	1	1	1	1	1	1	1	1	1
	11	1	1	1	1	1	1	1	1	1	1	1	1	1	1	1	1	1	1	1	1	1	1	1	1	1	1	1	1	1	1	1	1	1	1	1	1
	12	1	1	1	1	1	1	1	1	1	1	1	1	1	1	1	1	1	1	1	1	1	1	1	1	1	1	1	1	1	1	1	1	1	1	1	1

Replicate 8
