# Supplementary material for: CRISPR-Cas immunity leads to a coevolutionary arms race between Streptococcus thermophilus and lytic phage
Source: Philos Trans R Soc Lond B Biol Sci. 2019 Mar 25;374(1772):20180098. doi: 10.1098/rstb.2018.0098 (PMC6452269; doi:10.1098/rstb.2018.0098)
Supplement: Table S2 [file rstb20180098supp3.docx]

| **Replicate** | **Timepoint** | **Locus** | **Start** | **End** | **Sequence** | **N** | **Gene description** | **GeneID** |
| --- | --- | --- | --- | --- | --- | --- | --- | --- |
| 2 | 9 | CR1 | 647 | 676 | ATATCGTCCAGACTATCGCAGAATACTGAT | 11 | Hypothetical protein | 5176920 |
| 3 | 4 | CR1 | 871 | 900 | GTTTTAAGTGGTATTATTATATTATCGAAG | 1 | Intergenic region |  |
| 3 | 9 | CR1 | 871 | 900 | GTTTTAAGTGGTATTATTATATTATCGAAG | 10 | Intergenic region |  |
| 3 | 4 | CR1 | 1020 | 1049 | CATAGCTTACAATGCGGCTCTTAAAGCTGG | 4 | Terminase small subunit | 5176919 |
| 5 | 9 | CR1 | 2214 | 2185 | GGTCATCACCATTAACTAATCGAATAAGAT | 1 | Intergenic region |  |
| 3 | 9 | CR1 | 2821 | 2850 | TTTTTCGATGAGATATATCAGTACCGATGG | 1 | Terminase large subunit | 5176917 |
| 6 | 4 | CR1 | 10717 | 10746 | ACGAAGGCTTGGAAAACATTTGATGGTAAC | 12 | Tail protein | 5176903 |
| 6 | 9 | CR1 | 10717 | 10746 | ACGAAGGCTTGGAAAACATTTGATGGTAAC | 12 | Tail protein | 5176903 |
| 2 | 9 | CR1 | 16002 | 16031 | TATGGTGTCGAAACTTTTAAACGTTACAAT | 1 | Tail protein | 5176902 |
| 3 | 4 | CR1 | 20696 | 20725 | TGGAATAAGGTATCGTGCGTTTTGACAAGC | 1 | Antireceptor | 5176901 |
| 6 | 9 | CR1 | 22424 | 22395 | CACATGATCTACAACTAGGTCAAGATTGCT | 2 | Structural protein | 5176900 |
| 5 | 9 | CR1 | 23363 | 23334 | ATAGTTAACGCCTTTACACCGATCGAGGAA | 5 | Structural protein | 5176900 |
| 4 | 4 | CR1 | 23571 | 23600 | TTGTTAAAAGAAGCACTAGAGGTGATTTAC | 12 | Hypothetical protein | 5176899 |
| 4 | 9 | CR1 | 23571 | 23600 | TTGTTAAAAGAAGCACTAGAGGTGATTTAC | 12 | Hypothetical protein | 5176899 |
| 6 | 9 | CR1 | 25561 | 25531 | AAACAAAAAATCTTTGAAGTTTATGACATAC | 1 | Hypothetical protein | 5176893 |
| 5 | 9 | CR1 | 26669 | 26639 | GCATACCACAGGTATGACCAAAAACAAGAAA | 2 | Hypothetical protein | 5176889 |
| 2 | 9 | CR1 | 27003 | 27032 | AGATTTATAACATGGAAATTGACGATGAAA | 11 | Hypothetical protein | 5176888 |
| 6 | 9 | CR1 | 27003 | 27032 | TTTCATCGTCAATTTCCATGTTATAAATCT | 2 | Hypothetical protein | 5176888 |
| 3 | 9 | CR1 | 27358 | 27388 | AACACTCAAAGAGTTACTTAAATCTGGAAAG | 9 | Hypothetical protein | 5176888 |
| 6 | 9 | CR1 | 27410 | 27380 | CTGGAAAGCATATTGAGGGAGCTACTCTTG | 2 | Hypothetical protein | 5176888 |
| 3 | 4 | CR1 | 28744 | 28773 | AGCTATAGTATATACACATAGCGTAGAAGC | 3 | Helicase | 5176886 |
| 6 | 9 | CR1 | 29124 | 29095 | CAAAATAATTGTGGAAAATCACTGGTAAGT | 1 | Helicase | 5176886 |
| 6 | 9 | CR1 | 29407 | 29378 | AACAACCATTATTTGGGTTGGCCCGAATAT | 1 | Helicase | 5176886 |
| 3 | 9 | CR1 | 29618 | 29647 | TTTTCCGTCTTCTTTTTTAGCAAAGATACG | 1 | Hypothetical protein | 5176885 |
| 4 | 9 | CR1 | 29618 | 29647 | TTTTCCGTCTTCTTTTTTAGCAAAGATACG | 11 | Hypothetical protein | 5176885 |
| 5 | 9 | CR1 | 29618 | 29647 | TTTTCCGTCTTCTTTTTTAGCAAAGATACG | 5 | Hypothetical protein | 5176885 |
| 2 | 1 | CR1 | 29894 | 29923 | TTATGGAGATGGTTGATTACGCAATCAACT | 12 | Replication protein | 5176884 |
| 3 | 9 | CR1 | 30017 | 29988 | GCGACTGTTTGGTGGTTACTGACTTTTGCT | 8 | Replication protein | 5176884 |
| 1 | 9 | CR1 | 31582 | 31611 | CTCAGTCGTTACTGGTGAACCAGTTTCAAT | 12 | Primase | 5176883 |
| 3 | 9 | CR1 | 32557 | 32584 | GTATCAAACCAACGTCCATCAGCCATTC | 1 | Hypothetical protein | 5176882 |
| 7 | 9 | CR3 | 32554 | 32584 | TCAGAATGGCTGATGGACGTTGGTTTGATAC | 11 | Hypothetical protein | 5176882 |
| 7 | 4 | CR3 | 32554 | 32584 | TCAGAATGGCTGATGGACGTTGGTTTGATAC | 12 | Hypothetical protein | 5176882 |
| 8 | 9 | CR1 | 32707 | 32736 | TGAAAAAACGAGGAGCACTCGTAGGAGTGG | 12 | Hypothetical protein | 5176882 |
| 4 | 9 | CR1 | 33769 | 33799 | CTGCGTGGAACTGTCAGAACATAGTAGACTG | 1 | Hypothetical protein | 5176878 |
| 6 | 9 | CR1 | 33877 | 33848 | TGGTAACTGAAAGGTCAGTGGAACGGCACG | 1 | Hypothetical protein | 5176878 |
| 6 | 9 | CR1 | 33998 | 33968 | GTACAGAATTATTGAGGAGTTTATTGAACCT | 5 | Hypothetical protein | 5176878 |
| 3 | 4 | CR1 | 34587 | 34616 | AGCCTAGATAGCGAAGTTGATCGTATCTAT | 2 | Hypothetical protein | 5176877 |
