# Supplementary material for: CRISPR-Cas immunity leads to a coevolutionary arms race between Streptococcus thermophilus and lytic phage
Source: Philos Trans R Soc Lond B Biol Sci. 2019 Mar 25;374(1772):20180098. doi: 10.1098/rstb.2018.0098 (PMC6452269; doi:10.1098/rstb.2018.0098)
Supplement: Table S3 [file rstb20180098supp4.docx]

| **Primer name** | **Primer sequence** | **Annealing position** |
| --- | --- | --- |
| 1F | ACCAGTTGGAAGGAAAAGCTCT | 29733 |
| 2F | AGACGGCTCATTTGTGGGTT | 911 |
| 3F | CAACAGCAGCAAACACTGGG | 20496 |
| 4F | GACAACGGAAACATCGCACC | 28574 |
| 5F | GCACACCCTAACTGCGTCAT | 34377 |
| 6F | TGGACCCCTAGCGGAAGTTA | 23291 |
| 7F | ATGGCATGTCTAGCGCTCTC | 10532 |
| 8F | GTGGGCACTGCTAAGAGTGT | 32412 |
| 9F | TCAATCGGCTTTGAACGCAC | 726 |
| 10F | CTGAACGTTTCGGTCTTGCC | 31485 |
| 11F | GAATATCCACGCTGGCGAGA | 474 |
| 12F | TTGGGGTCGTCCTCACATTG | 15809 |
| 13F | AATTGAAGCACATCGGGGGA | 26835 |
| 14F | AGCAAGGAAACTGACTGGCA | 2625 |
| 15F | GCAGCGCTTGCGATTAGTAT | 27206 |
| 16F | GTCCGATGTGTGGTCACGAA | 29208 |
| 17F | TCAACCATTGGGCAGACGAA | 33405 |
| 26F | AAGAGCGGTGTCCTCGAAAG | 32230 |
