# Supplementary material for: CRISPR-Cas immunity leads to a coevolutionary arms race between Streptococcus thermophilus and lytic phage
Source: Philos Trans R Soc Lond B Biol Sci. 2019 Mar 25;374(1772):20180098. doi: 10.1098/rstb.2018.0098 (PMC6452269; doi:10.1098/rstb.2018.0098)
Supplement: Table S4 [file rstb20180098supp5.pdf]

Timepoint	Replicate	Phage ID	SNP in protospacer	SNP location 1	SNP location 2	SNP location 3
1	2	5	1	29896		
1	2	6	1	29899		
4	2	1	1	29899		
4	2	2	0			
4	2	3	0			
4	2	4	0			
4	2	5	0			
4	3	1	0			
4	3	2	1	1039		
4	3	3	0			
4	3	4	1	1039		
4	3	10	1	1027		
4	3	11	0			
4	6	3	1	10724		
4	6	4	1	10724		
4	6	5	1	10724		
4	6	6	1	10724		
4	6	9	1	10724		
4	7	1	1	32580		
4	7	2	1	32583		
4	7	12	1	32580		
4	8	1	0			
4	8	6	0			
4	8	8	0			
9	1	7	1	31615		
9	1	8	1	31615		
9	2	1	1	682		
9	2	5	1	682		
9	3	2	1	893	1014	
9	3	3	0			
9	3	9	1	893	1023	29983
9	3	10	0			
9	3	11	1	893	1024	1027
9	3	12	1	893	1023	
9	4	2	0			
9	4	3	1	29614	29626	
9	4	4	1	29614		
9	4	6	1	29614		
9	4	8	1	29614	29623	
9	6	1	1	10724		
9	6	8	1	10724		
9	6	9	1	10724		
9	7	1	1	32587		

9	7	2	1	32587		
9	7	5	1	32587		
9	7	6	1	32583		
9	7	9	1	32587		
9	7	10	0			
9	8	1	1	32747		
9	8	2	1	32747		
Ancestral	NA	1	0			
Ancestral	NA	2	0			
Ancestral	NA	3	0			
Ancestral	NA	4	0			
Ancestral	NA	5	0			
Ancestral	NA	6	0			
Ancestral	NA	7	0			
Ancestral	NA	8	0			
Ancestral	NA	9	0			
Ancestral	NA	10	0			
Ancestral	NA	11	0			
Ancestral	NA	12	0			
Ancestral	NA	13	0			
Ancestral	NA	14	0			
Ancestral	NA	15	0			
Ancestral	NA	16	0			
Ancestral	NA	17	0			
Ancestral	NA	18	0			
